# C/EBP*β* LIP augments cell death by inducing osteoglycin

**DOI:** 10.1038/cddis.2017.155

**Published:** 2017-04-06

**Authors:** Rina Wassermann-Dozorets, Menachem Rubinstein

**Affiliations:** 1 Department of Molecular Genetics, The Weizmann Institute of Science, Rehovot, Israel

## Abstract

Many types of tumor cell are devoid of the extracellular matrix proteoglycan osteoglycin (Ogn), but its role in tumor biology is poorly studied. Here we show that RNAi of Ogn attenuates stress-triggered cell death, whereas its overexpression increases cell death. We found that the transcription factor C/EBP*β* regulates the expression of Ogn. C/EBP*β* is expressed as a full-length, active form (LAP) and as a truncated, dominant-negative form (LIP), and the LIP/LAP ratio is positively correlated with the extent of cell death under stress. For example, we reported that drug-resistant tumor cells lack LIP altogether, and its supplementation abolished their resistance to chemotherapy and to endoplasmic reticulum (ER) stress. Here we further show that elevated LIP/LAP ratio robustly increased Ogn expression and cell death under stress by modulating the mitogen-activated protein kinase/activator protein 1 pathway (MAPK/AP-1). Our findings suggest that LIP deficiency renders tumor cell resistant to ER stress by preventing the induction of Ogn.

The endoplasmic reticulum (ER) is a large membrane-enclosed cellular organelle, found in all eukaryotes.^1^ Normal ER function is essential for many cellular processes, such as synthesis, modification and delivery of proteins to sites within the cell, on the cytoplasmic membrane and the extracellular space, synthesis of lipids and storage of calcium ions.^2^ Multiple physiological and pathophysiological stresses, such as protein over-production, hypoxia, glucose deprivation and aberrations in calcium ion regulation, lead to accumulation of unfolded proteins, thereby causing ER stress. To sense and respond to ER stress, eukaryotic cells have a conserved group of signal transduction pathways, collectively termed the unfolded protein response (UPR).^3^ The UPR constitutes of three major signaling pathways, initiated by activation of the ER membrane proteins IRE1, PERK and ATF6. Initially, the UPR is aimed at re-establishing cellular homeostasis.^4^ However, extensive ER stress redirects the UPR toward cell death by activating several mechanisms,^5^ including induction of pro-death transcription factor CCAAT/enhancer-binding protein homologous protein (CHOP).^2^ CHOP then triggers oxidative stress and DNA damage, resulting in cell death.^6^ CHOP also triggers apoptosis directly by repressing transcription of the anti-apoptotic Bcl-2 proteins.^2^

Mitogen-activated protein kinases (MAPKs) are serine–threonine kinases that mediate intracellular signaling associated with a broad range of cellular activities. The major MAPK groups are extracellular signal-regulated kinase 1/2 (ERK1/2), c-Jun N-terminal kinases 1/2/3 (JNK1/2/3) and stress-activated protein kinases *α/β/γ/δ* (SAPK/p38*α/β/γ/δ*).^7^ The MAPK signaling pathways are involved in regulation of the UPR. Whereas ERK1/2 activation is IRE1-dependent and usually promotes cell survival, JNK and p38 activation promotes ER stress-triggered cell death. The IRE1/ASK1 and the PERK/CHOP axes activate the JNK pathway, promoting cell death by inducing death receptors and their ligands, by direct interaction and phosphorylation of Bcl-2 family proteins, and by induction and activation of activator protein 1 (AP-1) family of transcription factors. The p38 pathway is activated by the IRE1/ASK1 axis, leading to cell death by increasing the transcriptional activity of CHOP and ATF6, by cell cycle arrest, Bim phosphorylation and AP-1 activation.^8^

ER stress triggers the expression of the transcription factor C/EBP*β* as part of the UPR.^9, 10^ C/EBP*β* is expressed in normal and tumor cells as a full-length, active form (LAP) and a truncated, natural dominant-negative form (LIP), generated by translation initiation from an internal AUG site of the single *C/EBPβ* mRNA.^11^ The LIP/LAP ratio is positively correlated with the extent of mouse melanoma cell death under ER stress.^12^ More recently, we found that both constitutive and selected drug-resistant tumor cells lack LIP altogether and supplementation of LIP restores their sensitivity to chemotherapy and to triggers of ER stress.^13^ One function of LIP is to assist in nuclear translocation of CHOP during ER stress.^14^ Other studies reported LIP-mediated inhibition of the c-Jun coactivator Jab1, a novel candidate oncogene highly expressed in breast carcinoma.^15^ In addition, LIP suppresses the promoter activity of a tumor suppressive miR-145.^16^ However, the mechanism by which LIP causes cell death under stress is not completely known.

To further study the role of elevated LIP/LAP ratio in ER stress-triggered cell death, we used B16 melanoma subline and JC mammary gland cancer cells, constitutively expressing doxycycline (Doxy)-inducible LIP. Our data show that elevated LIP/LAP ratio augments cell death mainly by activating the MAPK/AP-1 pathway. We further show that elevated LIP induces the extracellular matrix proteoglycan osteoglycin (Ogn), which in turn augments ER stress-triggered cell death. It is, therefore, likely that LIP deficiency renders tumor cells resistant to ER stress by preventing the induction of Ogn.

## Results

### LIP signals through the MAPK and the JAK/STAT3 pathways under ER stress

To confirm previously reported effect of LIP on cell death, we induced LIP expression with Doxy in B16-F10.9-4 and JC TetON LIP cells and then treated the cells with three common ER stress inducers tunicamycin (Tm), thapsigargin (Tg) and brefeldin A (BFA). Treatment with Doxy alone only slightly decreased cell viability, whereas a combined treatment with Doxy and each of the ER stress inducers resulted in stronger effect (Supplementary Figures 1a and b). To find out whether the effect on cell viability is due to activation of cell death, we evaluated three possible cell death pathways. Tm triggered necrosis and apoptosis to the same extent in control cells and following induction of LIP, as indicated by HMGB1 release and by cleavage of caspase-3, respectively. The level of LC3B was not significantly changed, indicating that autophagy is not involved in Tm-triggered cell death (Supplementary Figure 1c). Induction of LIP did not elicit these death markers in the absence of ER stressors and marginally inhibited cell proliferation (Supplementary Figures 1c and d). Taken together, these findings demonstrate that LIP augments ER stress-triggered cell death.

**Figure 1:**
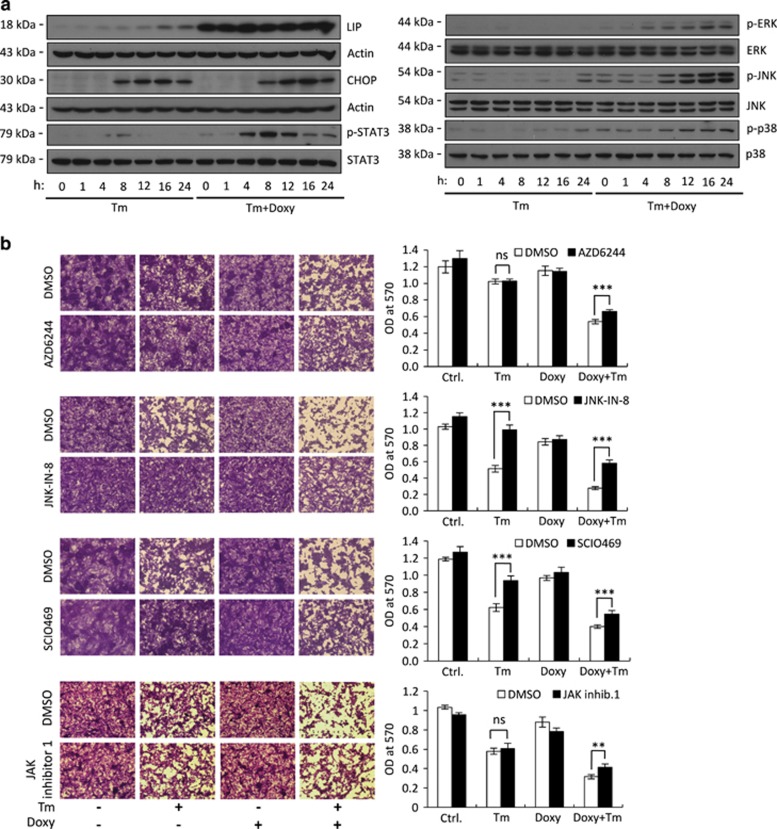
LIP augments ER stress-triggered cell death by activating the MAPK pathway. (**a**) Immunoblot of the indicated proteins in total cell extract of F10.9-4 cells pretreated with vehicle or Doxy, followed by vehicle or Tm for the indicated times. *N*=2. (**b**) Crystal violet staining of F10.9-4 cells pretreated with vehicle or Doxy, followed by vehicle or Tm, in the presence or absence of the ERK1/2 inhibitor AZD6244, the JNK inhibitor JNK-IN-8, the p38 inhibitor SCIO469 and the JAK inhibitor 1. *N*=2–3, **P<0.01, ***P<0.001; NS, not significant

To identify death-mediating signaling pathways regulated by LIP, we increased the LIP/LAP ratio in F10.9-4 cells by LIP induction and then triggered ER stress using Tm. RNA was then isolated and subjected to expression array analysis. Genes associated with a wide range of cellular processes, including apoptosis, necrosis, angiogenesis, survival, growth, metastasis and migration, were affected by elevated LIP under ER stress (Supplementary Table 1). Two groups of transcripts implicated the MAPK and the Janus kinase/signal transducers and activators of transcription 3 (JAK/STAT3) as possible signaling pathways involved in LIP-mediated augmentation of cell death. To further study these pathways, we induced LIP expression as above followed by treatment with inducers of ER stress. Immunoblotting revealed extensive LIP-mediated activation of the three MAPK pathway components ERK1/2, JNK and p38, as well as that of STAT3 under stress, as seen by the appearance of their phosphorylated forms. JNK and p38 were activated during the cell death phase of the UPR, characterized by CHOP induction. In contrast, LIP-mediated phosphorylation of ERK and STAT3 began before CHOP induction (Figure 1a, Supplementary Figure 2).

### MAPK is the major death-promoting pathway activated by LIP

Next, we determined whether LIP-mediated activation of the MAPK and JAK/STAT3 pathways augmented cell death. To inhibit three MAPK pathways, we used highly specific inhibitors, evaluating their efficacy by immunoblotting (Supplementary Figure 3). The JNK and p38 inhibitors, JNK-IN-8 and SCIO469, but not the ERK1/2 inhibitor AZD6244, significantly attenuated ER stress-triggered cell death (Figure 1b), in line with the fact that Tm alone did not activate ERK1/2 (Figure 1a). Nevertheless, all three inhibitors significantly attenuated the death-promoting effect of LIP (Figure 1b). Similar results were seen with three other commonly used MAPK inhibitors. The ERK1/2 inhibitor U0126, JNK inhibitor SP600125 and p38 inhibitor SB203580 effectively inhibited MAPK activity despite demonstrating some off-target effects (Supplementary Figure 4). All three inhibitors markedly attenuated LIP augmentation of ER stress-triggered cell death (Supplementary Figure 5a and Supplementary Figures 6 and 7). Of note, treatment with each MAPK inhibitor alone did not prevent cell death completely, but a treatment of the cultures with a combination of all three inhibitors completely prevented Tm-triggered cell death and almost completely prevented the death-promoting effect of LIP (Supplementary Figure 5b). In contrast with the strong effect of MAPK inhibitors, the JAK inhibitor I only slightly attenuated the LIP-mediated augmentation of ER stress-triggered cell death (Figure 1b). Taken together, these findings indicate that the MAPK pathway has a central role both in ER stress-triggered cell death and in its augmentation by LIP.

### The role of AP-1 in augmentation of cell death by LIP

**Figure 2:**
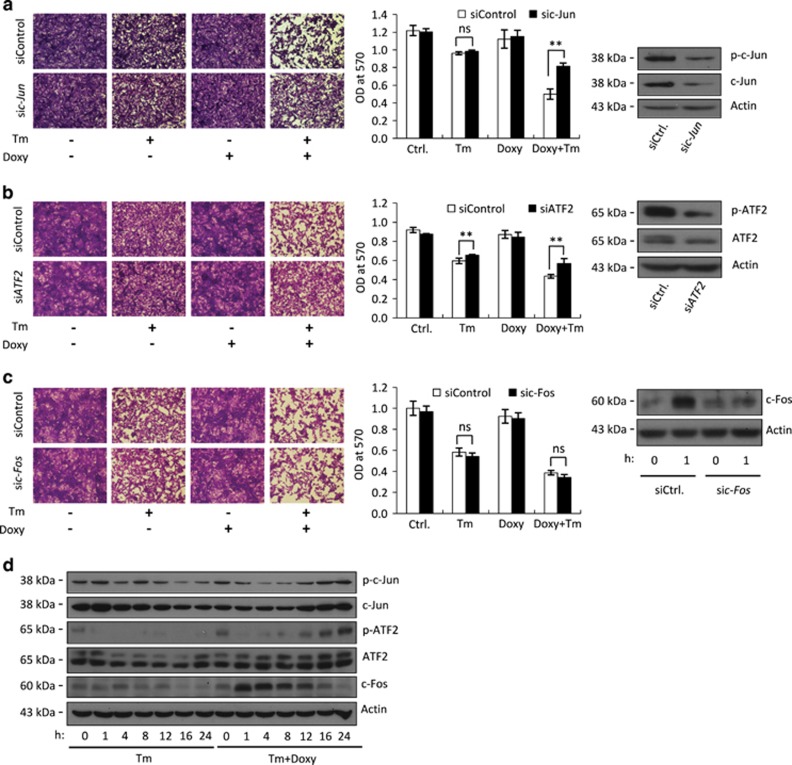
The role of AP-1 in augmentation of cell death by LIP. (**a–c**) Crystal violet staining of F10.9-4 cells transfected with the indicated siRNA at time=0, treated with vehicle or Doxy at time=24 h, followed by vehicle or Tm at time=48 h. *N*=2–3, **P<0.01; NS, not significant. Silencing efficacy was evaluated by immunoblotting of total cellular proteins isolated immediately after the Tm treatment (si*c-Jun* and si*ATF2*) or immediately and after 1 h (si*c-Fos*) of Tm treatment. (**d**) Immunoblot of the indicated proteins in total cell extract of F10.9-4 cells pretreated with vehicle or Doxy, followed by vehicle or Tm for the indicated times

The MAPK pathways activate the transcription factor AP-1, which is a homo- or heterodimer of c-Jun, c-Fos and ATF2.^17, 18^ Knockdown of either *c-Jun* or *ATF2* mRNA but not *c-Fos* mRNA abolished the LIP-mediated augmentation of ER stress-triggered cell death (Figures 2a–c, Supplementary Figure 8). LIP upregulated the early expression of c-Fos and the late expression of c-Jun and ATF2 under ER stress. Triggering ER stress at low LIP levels was not sufficient for activating AP-1, as it reduced the phosphorylation of ATF2 and c-Jun and reduced the level of c-Fos below their basal level, suggesting that LIP is required for AP-1 reactivation (Figure 2d, Supplementary Figure 2). C-Jun and ATF2 are usually phosphorylated and then activated by JNK and p38.^17, 18, 19^ The JNK inhibitor SP600125 reduced c-Jun and ATF2 phosphorylation, thereby reducing their activation at high LIP levels and under ER stress (Supplementary Figure 4b). Double knockdown of *c-Jun* and *ATF2* resulted in a lower effect on cell death than SP600125 treatment, indicating that there are additional cell death-promoting mediators downstream of JNK (Supplementary Figure 9). In contrast, the ERK inhibitor U0126 and the p38 inhibitor SB203580 prevented the dephosphorylation of c-Jun and ATF2, thereby increasing their activation (Supplementary Figures 4a and c). These results indicate a tight regulation of pro-death AP-1 transcription factors by all three MAPKs.

### LIP acts independently of several known UPR mediators and MAPK/AP-1 activators

MAPKs are activated by the IRE1 and PERK axes of the UPR,^8^ prompting us to study the possible involvement of LIP in activating these two arms of the UPR. The level of both ER stress-induced markers CHOP and BiP was not affected by LIP under stress (Supplementary Figure 10a). Similarly, LIP did not increase the phosphorylation of ASK1, a downstream target of IRE1 (Supplementary Figure 10b). These results show that LIP acts independently of these mediators of the UPR. The MAPK pathway is also activated by small GTPases from the Ras/Rho family.^7^ Similarly, inhibition of Ras by farnesyl thiosalicylate, of CDC42 by ML141 and of Rac1 by NSC23766 did not affect ER stress-triggered cell death, either without or with induction of LIP (Supplementary Figure 11). Rac1 and CDC42 activate MAPK pathways independently. Their concurrent inhibition by ML141 and NSC23766 did not attenuate cell death either (Supplementary Figure 11b). Taken together, these findings indicate that LIP augments ER stress-triggered cell death independently of Ras, Rac1 and CDC42.

The JAK/STAT are activated by many different stimuli, including cytokines and growth factors. These agents trigger receptor-mediated trans-phosphorylation of JAK, leading to phosphorylation of both the receptors and their associated STATs. In addition to the classical STAT3 activation, receptor phosphorylation allows parallel activation of other cascades, including Ras/MAPK, IRS and PI3K.^20^ This fact prompted us to check the role of the JAK/STAT3 pathway in LIP-mediated MAPK/AP-1 activation. In line with the minimal effect of JAK inhibition on cell survival, it also had no effect on activation of the components of MAPK/AP-1 axis (Supplementary Figure 12). These results suggest that LIP activates the MAPK/AP-1 pathway independently of JAK/STAT3 activation.

### ER stress induces Ogn

**Figure 3:**
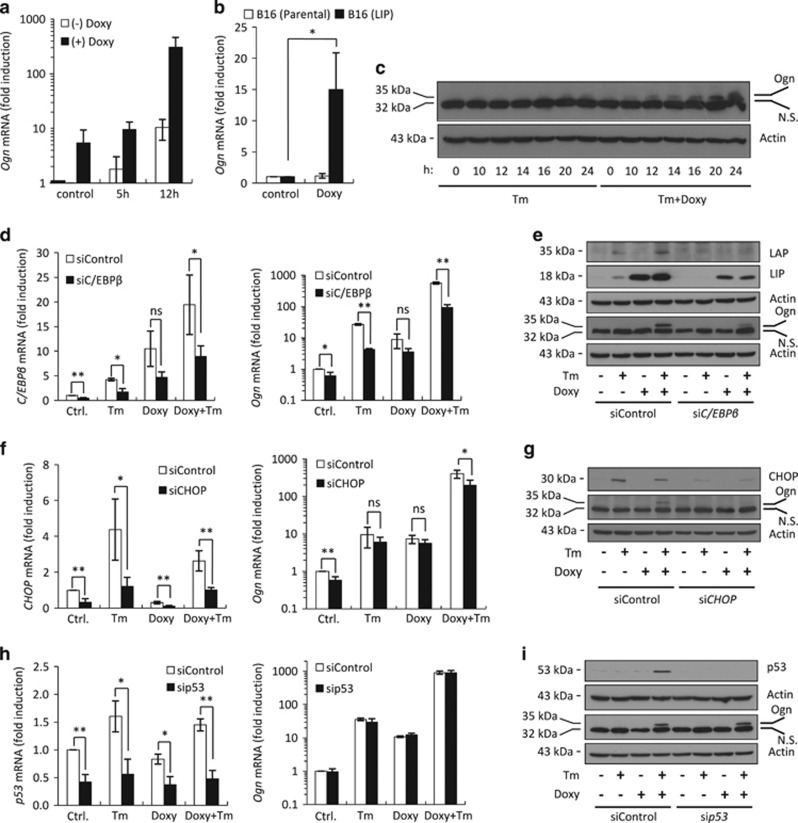
ER stress induces Ogn expression. (**a**) qRT-PCR of *Ogn* mRNA isolated from F10.9-4 cells pretreated with vehicle or Doxy, followed by vehicle or Tm for the indicated times. (**b**) qRT-PCR of *Ogn* mRNA isolated from parental B16-F10 cells and from B16-F10.9-4 cells treated with vehicle or Doxy. *N*=2, *P<0.05. (**c**) Immunoblot of Ogn in total cell extract of F10.9-4 cells pretreated with vehicle or Doxy, followed by vehicle or Tm for the indicated times. Ogn appears as a faint band above the non-specific (N.S.) band. (**d, f** and **h**) qRT-PCR of the indicated mRNA isolated from F10.9-4 cells transfected at time=0 with control siRNA, *C/EBPβ*-specific siRNA (**d**), *CHOP*-specific siRNA (**f**) or *p53*-specific siRNA (**h**), treated at time=24 h with vehicle or Doxy, followed by vehicle or Tm at time=48 h. RNA was isolated 24 h after Tm treatment. *N*=2, *P<0.05, **P<0.01; NS, not significant. (**e, g** and **i**) Immunoblot of the indicated proteins in total cell extracts of F10.9-4 cells treated as described in (**d, f** and **h**). *N*=1–2; NS, nonspecific band

We then looked for possible target genes of the LIP-activated MAPK/AP-1 pathway. The expression array analysis revealed that upregulation of LIP followed by 12 h of ER stress-induced *Ogn* mRNA expression by 10-fold (*P*=3.38E−09) compared with ER stress alone (Supplementary Table 2). The *Ogn* promoter contains three conserved AP-1-binding sites,^21^ prompting us to study *Ogn* as a target gene downstream to LIP/MAPK/AP-1. Using quantitative real-time PCR (qRT-PCR), we found that ER stress-induced *Ogn* mRNA by 10-fold and 300-fold at low and high LIP, respectively (Figure 3a). Induction was LIP specific, as no induction was seen upon treatment of the parental B16-F10 cells with Doxy (Figure 3b). At the protein level, ER stress-induced a rather low level of Ogn at low LIP, which was greatly increased at high LIP (Figure 3c, Supplementary Figure 13a). In an inverse experiment, knockdown of *C/EBPβ* mRNA by specific RNAi under ER stress downregulated the expression of *Ogn* mRNA and protein, thereby further confirming the expression array data (Figures 3d and e). Knockdown by RNAi of the ER stress-induced CHOP reduced the expression of Ogn as well (Figures 3f and g). ER stress induces p53 and independent studies reported that p53 activates the *Ogn* promoter.^21, 22, 23^ However, in our hands knockdown of p53 had no effect on the level of Ogn expression (Figures 3h and i).

**Figure 4:**
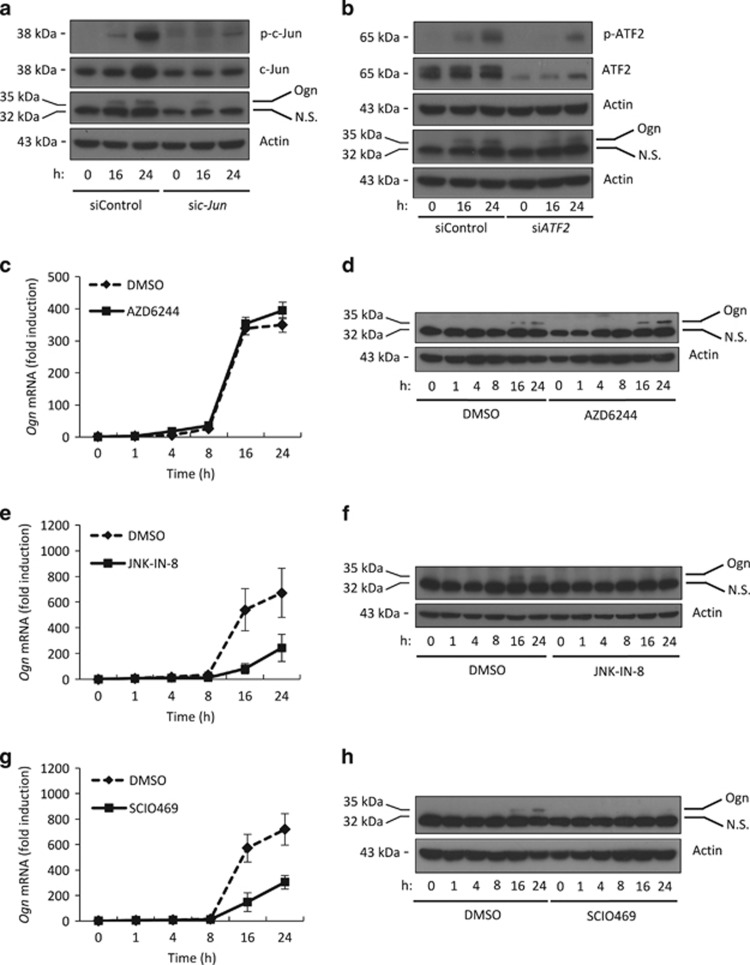
The MAPK/AP-1 axis regulates Ogn expression. (**a** and **b**) Immunoblot of the indicated proteins in total cell extracts of F10.9-4 cells transfected at time=0 with control siRNA, *c-Jun*-specific siRNA (**a**), or *ATF2*-specific siRNA (**b**), treated with vehicle or Doxy at time=24 h and vehicle or Tm at time=48 h. Cell extracts were isolated 0, 16 and 24 h after Tm treatment. (**c, e** and **g**) qRT-PCR of *Ogn* mRNA isolated from F10.9-4 cells pretreated with vehicle or Doxy, followed by vehicle or Tm for the indicated times, in the presence or absence of the ERK1/2 inhibitor AZD6244 (**c**), the JNK inhibitor JNK-IN-8 (**e**) or the p38 inhibitor SCIO469 (**g**). (**d, f** and **h**) Immunoblot of Ogn in total cell extracts of F10.9-4 cells treated as described in (**c, e** and **g**). NS, nonspecific band

We then examined whether LIP regulates Ogn expression by activation of MAPK/AP-1. Induction of Ogn was AP-1 dependent, as it was reduced by knockdown of c-Jun. However, knockdown of ATF2 did not affect Ogn expression, suggesting that an AP-1 consisting of a c-Jun homodimer or a heterodimer of c-Jun with an unidentified partner regulates Ogn expression (Figures 4a and b). Consistent with this observation, induction of Ogn was downregulated by SP600125 and upregulated by U0126 and SB203580 (Supplementary Figure 14). The highly specific inhibitor AZD6244 increased, whereas JNK-IN-8 inhibited Ogn expression, confirming the role of ERK1/2 and JNK in the regulation of Ogn expression (Figures 4c–f). Treatment with SCIO469 resulted in the opposite effect on Ogn expression (Figures 4g and h), indicating that results obtained with SB203580 inhibitor may be due to its off-target effect. Taken together, these results suggest that LIP promotes Ogn expression by JNK at the MAPK level, and by c-Jun at the AP-1 level.

### Ogn is involved in ER stress-triggered cell death

**Figure 5:**
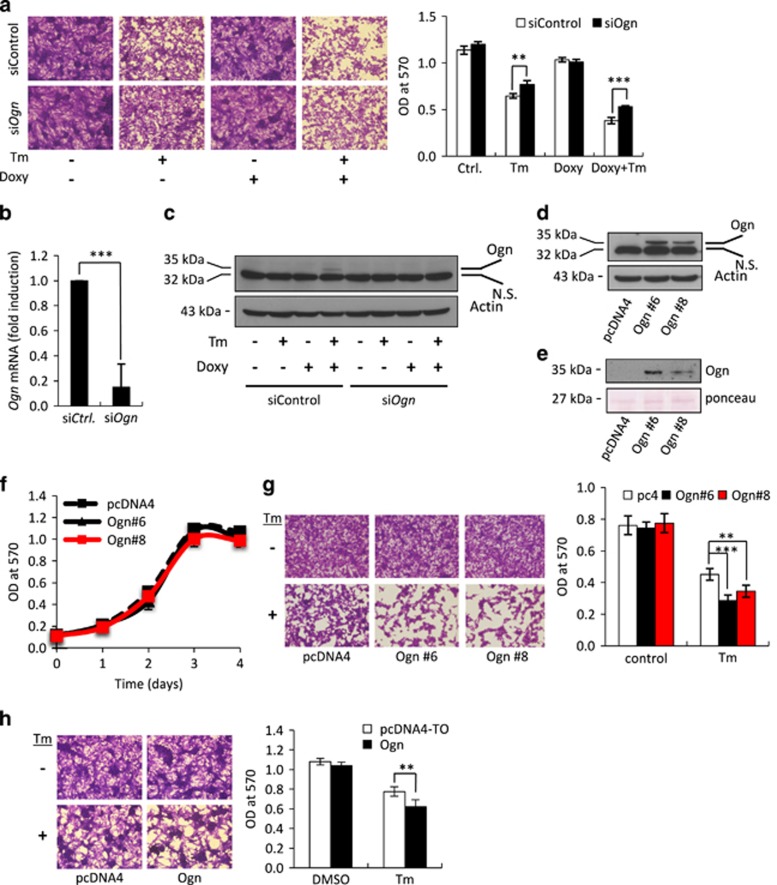
Ogn augments ER stress-triggered cell death. (**a**) Crystal violet staining of F10.9-4 cells transfected at time=0 with control siRNA or *Ogn*-specific siRNA, treated with vehicle or Doxy at time=24 h, followed by vehicle or Tm at time=48 h. *N*=10, **P<0.01, ***P<0.001. (**b**) qRT-PCR of *Ogn* mRNA in extracts F10.9-4 cells transfected at time=0 with control siRNA or *Ogn*-specific siRNA. Total mRNA was isolated at time=48 h. *N*=2, ***P<0.001. (**c**) Immunoblot of Ogn in total cell extracts of F10.9-4 cells treated as described in (**a**). *N*=4, NS, nonspecific band. (**d**) Immunoblot of Ogn in total cell extracts of B16-F10 cells stably transfected with pcDNA4-TO or with Ogn expression vector (clones #6 and #8). (**e**) Immunoblot of Ogn in serum-free culture medium of the indicated clones, isolated after 3 days and concentrated 10-fold. (**f**) Cell growth rate of B16-F10 Ogn-expressing clones and pcDNA4-TO-transfected clone. *N*=3. (**g**) Crystal violet staining of B16-F10 Ogn-expressing clones and pcDNA4-TO-transfected clone, treated with vehicle or Tm and then stained. *N*=4, **P<0.01, ***P<0.001. (**h**) Crystal violet staining of parental B16-F10 cells treated with vehicle or Tm, in the presence of medium collected from B16-F10 Ogn-expressing clone or pcDNA4-TO-transfected clone. *N*=3, **P<0.01

We then studied the possible role of Ogn in ER stress-triggered cell death. Specific knockdown of *Ogn* mRNA, both at low or high LIP level, attenuated ER stress-triggered cell death (Figures 5a–c). To test the effect of Ogn overexpression on ER stress-triggered cell death, B16-F10 cells were stably transfected with either control pcDNA4-TO vector or with pcDNA4-TO vector encoding m*Ogn*. Two clones, Ogn #6 and Ogn #8, showing the highest level of Ogn expression (Figures 5d and e) were chosen for further study. The high Ogn expression in these clones had no effect on their proliferation (Figure 5f). In contrast, both clones were significantly more sensitive than control cells to ER stress-triggered cell death following Tm (Figure 5g). The same results were obtained in JC TetON LIP cells treated with all three ER stress inducers (Supplementary Figures 13b and c) and upon using BFA and Tg in F10.9-4 cells (Supplementary Figures 12d–k). Hence, we conclude that the role of Ogn in cell death is rather general and not reagent or cell type specific.

As Ogn is an extracellular matrix protein, we asked if its secreted form mediates the death-promoting effect of Ogn. Indeed, we found that exposing parental B16-F10 cells to Ogn-containing media augmented Tm-triggered cell death, thereby demonstrating that it exerts its action on an extracellular target (Figure 5h).

To further study the regulation of Ogn by the JNK/c-Jun axis as demonstrated in Figure 4a, we performed knockdown of *Ogn* mRNA in B16 cells and in parallel treated them with the JNK inhibitor SP600125. The combined treatment did not result in further inhibition of cell death, indicating that Ogn belongs to the JNK pathway (Supplementary Figure 15a). Similarly, attenuation of cell death by double knockdown of *c-Jun* and *Ogn* mRNA was not additive (Supplementary Figure 14b). Taken together, these findings confirm that Ogn is mainly regulated by the JNK pathway and is the main effector for c-Jun-induced cell death. Although ATF2 does not regulate Ogn expression (Figure 4b), double knockdown of *ATF2* and *Ogn* mRNA did not lead to increased attenuation of cell death (Supplementary Figure 15c), suggesting that both ATF2 and Ogn may regulate the same downstream pathways.

Although we have demonstrated the pro-death effect of Ogn not much data exist as to a possible downstream action of Ogn. Several studies reported cooperation between Ogn and TGF*β*, which may explain its role in augmenting cell death.^24, 25^ However, in our hands knockdown of *Ogn* mRNA did not affect both the canonical and the non-canonical pathways of TGF*β* activation, as Smad2/3 and MAPK phosphorylation was not affected (Supplementary Figure 16). These results suggest that LIP-induced Ogn augments cell death independently of TGF*β*.

## Discussion

**Figure 6:**
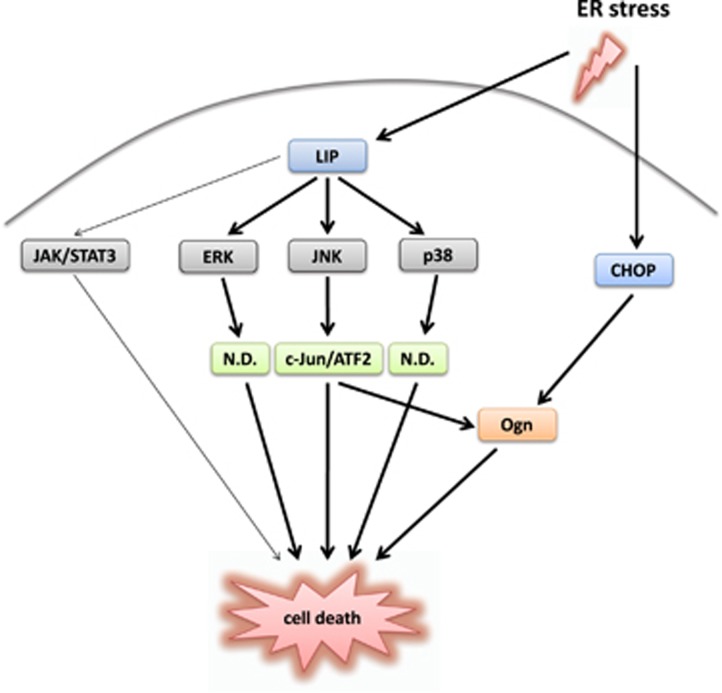
A scheme showing the LIP-mediated pathways following ER stress. ER stress increases Ogn expression by induction of C/EBP*β* LIP and CHOP. LIP activates the JAK/STAT3 pathway and three MAPK members ERK, JNK and p38. These, in turn, augment cell death by several mechanisms, including activation of AP-1 and induction of Ogn by c-Jun. ND, not determined

C/EBP*β* LIP, a truncated form of C/EBP*β*, was previously shown to promote cell death.^12, 13^ As LIP lacks activation domains, it is considered as a natural dominant-negative form of C/EBP*β*. Apart from competitive inhibition of C/EBP*β* LAP, not much is known about its mechanism of action.^11^ Our findings highlight a rather complex signaling pathway triggered by LIP, which augments ER stress-triggered cell death (Figure 6). This pathway involves mainly activation of MAPKs, of which JNK promotes cell death through activation of c-Jun/ATF2, whereas p38 and ERK1/2 trigger cell death by a pathway independent of c-Jun and ATF2. In fact, p38 and ERK1/2 seem not just to bypass but even to divert the signaling cascade from c-Jun/ATF2 to alternative pathways, as inhibitors of p38 and ERK increased phosphorylation and hence activation of c-Jun and ATF2 (Supplementary Figures 4a and c). The exact nature of these putative alternative pathways remains to be established. Possible p38-activated candidates include p53, Bim, CHOP and ATF6.^26, 27, 28, 29^ In the case of ERK1/2, more than 150 targets have been identified.^30^ Among them are various death receptors (Fas, DR4 and DR5) and death ligands (TNF*α*, FasL), Bcl-2 family members, p53 and Elk1.^30, 31, 32^ Our finding that ERK1/2 has a role in cell death may be associated with its subcellular localization, as blocking its nuclear translocation induced apoptosis of melanoma cells.^33, 34^ Therefore, it is possible that LIP augments cell death by inhibiting nuclear translocation of ERK1/2. As the UPR mediators BiP, CHOP and ASK1, as well as small GTPases from the Ras/Rho family, were not involved in augmentation of cell death by LIP, the upstream LIP-activated regulator of MAPK remains to be identified. Possible candidates may include Mos, Tlp2, TAK1 and TAO1/2 kinases.^7^

Previous studies implicated the JAK/STAT3 pathway mainly in enhancing tumor growth,^35^ but recent studies also linked it with NOX1 and lysosome-mediated cell death.^36, 37^ We found that the JAK/STAT3 pathway is MAPK/AP-1-independent as no effect of JAK inhibitor on MAPK and AP-1 activation was observed. The almost complete inhibition of cell death by a combination of all three MAPK inhibitors is in line with the rather limited role of the JAK/STAT3 pathway in LIP augmentation of cell death.

Ogn is an extracellular matrix proteoglycan, belonging to the small leucine-rich protein (SLRP) family, whose role in cell biology is not well established.^38^ Several studies have shown loss of Ogn expression in the majority of cancer cell lines and tumors,^39, 40, 41^ suggesting its potential role as a tumor-suppressor gene. However, the precise role of Ogn in tumor development has not yet been elucidated. Here we have demonstrated the role of Ogn in ER stress-triggered cell death at both low and high LIP level. These results are in line with studies showing the involvement of other extracellular matrix proteins, such as type 1 collagen and fibronectin in cancer cell biology, where they activate intracellular signaling pathways.^42, 43, 44^ Our findings that Ogn expression is upregulated by C/EBP*β* LIP and CHOP are in line with a study demonstrating induction of Ogn mRNA expression by UV irradiation, as UV triggers ER stress.^21^ p53 was reported to activate the Ogn promoter.^21, 22, 45^ Our finding that p53 had no effect on *Ogn* mRNA and protein expression is in contrast to this report. Although the mouse B16-F10 melanoma cell line is reported to have wt p53,^45^ some point mutations during LIP inducible clone development may occur, leading to p53 dysfunction. Our observation that LIP-induced Ogn mainly through the JNK/c-Jun axis, whereas ERK1/2 inhibited Ogn expression is in line with the observation that ERK1/2 downregulated c-Jun. These results suggest a complex regulation of Ogn level by MAP kinases, where the ratio between the positive effect of JNK and the negative effect of ERK1/2 determine the impact of MAPK activation on induction of Ogn. Knockdown of Ogn attenuated cell death to a lesser extent than that obtained by inhibition of the MAPK pathways. Therefore, additional cell death mechanisms must exist on top of Ogn induction. Our findings indicate that Ogn acts independently of TGF*β* in augmenting B16 melanoma cell death. TNF*α* interacts with two other members of the SLRP family: biglycan and decorin.^46^ Hence, similar interactions with other TNF family members may explain the role of Ogn in triggering cell death.

We recently demonstrated that loss of LIP by lysosomal and proteasomal degradation is responsible for resistance of several cell lines to ER stress-triggered cell death, and supplementation of LIP reversed this resistance.^13^ Rapidly growing solid tumors endure lagging angiogenesis, leading to hypoxia, nutrient deprivation and accumulation of toxic metabolites. These pathophysiological conditions trigger ER stress and subsequent cell death.^5^ It is possible that lack of LIP-induced Ogn provides resistance from ER stress-triggered cell death, thereby providing a rationale for the absence of Ogn expression in cancer cell lines and tumors.^39, 40, 41^

In summary, we demonstrated that LIP augments ER stress-triggered cell death by activating three members of the MAPK family, each one further activating a distinct signaling cascade. Ogn, whose expression was highly increased by the LIP/JNK/c-Jun cascade, augments ER stress-triggered cell death. Loss of Ogn in several cell lines and tumors together with its ability to promote cell death, suggest its function as tumor-suppressor gene.

## Materials and methods

### Reagents

Tm (T7765), BFA (B7651), Tg (T9033), Doxy hyclate (D9891), ponceau (P7170-1L) and crystal violet solution (V-5265) were purchased from Sigma-Aldrich (Rehovot, Israel). Specific inhibitors: U0126 (sc-222395), SP600125 (sc-200635), SB203580 (sc-3533), JAK inhibitor I (sc-204021), NSC23766 (sc-204823), ML141 (sc-362768), FTS (sc-205322), SCIO469 (sc-361353), AZD6244 (sc-364613a) and JNK-IN-8 (sc-364745) were purchased from Santa Cruz Biotechnology (Dallas, TX, USA). Zeocin (ant-zn-1) was purchased from InvivoGen (San Diego, CA, USA). Primary antibodies to p-ERK1/2 (#4370), ERK1/2 (#4695), p-JNK (#9255), JNK (#9252), p38 (#9212), p-c-Jun (#9261), p-ATF2 (#9221), ATF2 (#9226), p-STAT3 (#9131), STAT3 (#9139), p-Smad2 (#3108), p-ASK1 (#3765), cleaved caspase-3 (#9661), p-p38 (#4511) and p53 (1C12) were purchased from Cell Signaling (Danvers, MA, USA). Antibodies to C/EBP*β* (C-19), CHOP (R-20), Ogn (G-1), Smad2 (YZ-13), ASK1 (H-300), c-Fos (H-125) and Smad4 (B-8) were purchased from Santa Cruz Biotechnology. Antibodies to p-Smad3 (ab51451) and Smad3 (ab28379) were purchased from Abcam (Cambridge, MA, USA). Antibodies to LC3B (L7543) and HMGB1 (H9537) were purchased from Sigma-Aldrich. Antibodies to c-Jun (610326) and BiP (610978) were purchased from BD Transduction Laboratories (San Jose, CA, USA). Actin antibody (#69100) was purchased from MP Biomedicals (Santa Ana, CA, USA). The secondary antibodies, goat anti-mouse IgG (H+L) (#115-035-062) and goat anti-rabbit IgG (H+L) (#111-035-144), were purchased from Jackson ImmunoResearch (West Grove, PA, USA).

### Cell culture

The murine B16 melanoma clone F10.9-4, stably transfected with inducible LIP was derived from the parental cell line B16-F10 (ATCC, CRL-6475; Manassas, VA, USA) and was kindly provided by M Revel.^47^ Ogn stably overexpressing clones, Ogn#6 and Ogn#8, were derived from the parental cell line B16-F10 as part of this study. All B16 melanoma cell lines were cultured in DMEM containing 10% FBS, 2 mM l-glutamine solution, 50 U/ml penicillin and 0.1 mg/ml streptomycin in humidified 8% CO_2_ incubator at 37 °C. Expression of LIP was induced by Doxy (2 *μ*g/ml) treatment for 24 h. ER stress was induced by treating cells for 18–24 h with Tm (0.25 *μ*g/ml), BFA (0.5 *μ*g/ml) or Tg (25 nM) unless otherwise stated. When necessary, specific inhibitors U0126 (20 *μ*M), SP600125 (40 *μ*M), SB203580 (40 *μ*M), JAK inhibitor I (1 *μ*M), ML141 (12.5 *μ*M), NSC23766 (50 *μ*M), FTS (50 *μ*M), AZD6244 (20 *μ*M), JNK-IN-8 (10 *μ*M) and SCIO469 (5 *μ*M) were added together with the ER stress inducer. The murine JC TetON LIP mammary gland cancer cells, stably transfected with inducible LIP was derived from the parental cell line JC (ATCC, CRL-2116) and was kindly provided by C Riganti.^13^ JC TetON LIP cells were cultured in RPMI containing 10% FBS, 2 mM l-glutamine solution, 50 U/ml penicillin and 0.1 mg/ml streptomycin in humidified 5% CO_2_ incubator at 37 °C. Expression of LIP was induced by Doxy (1 *μ*g/ml) treatment for 16 h. ER stress was induced by treating cells for 18–24 h with Tm (0.125 *μ*g/ml), BFA (0.125 *μ*g/ml) or Tg (25 nM) unless otherwise stated. Where indicated, the inhibitors U0126 (20 *μ*M), SP600125 (40 *μ*M), SB203580 (40 *μ*M) were added together with the ER stress inducer.

### Assay of cell viability

B16 cells (4 × 10^5^ cells/ml) or JC TetON LIP cells (2 × 10^5^ cells/ml) were cultured in 96-well plates and grown for 16–24 h before treatments. After the indicated treatments, cultures were stained with 0.5% crystal violet in 50% aqueous ethanol and photographed under a light microscope. Quantification of the relative survival of the cells was done by ELISA reader at 570 nm and shown in the graphs.

### Proliferation assay

B16 cells (7 × 10^4^ cells/ml) were cultured in 96-well plates. Where indicated, expression of LIP was induced by Doxy (2 *μ*g/ml). At the indicated times, the cultures were fixed and stained with 0.5% crystal violet in 50% aqueous ethanol and photographed under a light microscope. Quantification of the relative survival of the cells was done using ELISA reader at 570 nm.

### Production of Ogn containing media

B16 cells stably transfected with pcDNA4-TO-Ogn (clone #6) or with control pcDNA4-TO vector (4 × 10^5^ cells/ml) were cultured in 15 cm dishes. After 20 h, cells reached 80% confluence, the media were changed to fresh serum-free media. After 3 days, media were collected, concentrated twofold by ultrafiltration and supplemented with 10% serum. These media were used for the treatment of parental B16-F10 cells.

### Immunoblotting

B16 cells (4 × 10^5^ cells/ml) or JC TetON LIP cells (2 × 10^5^ cells/ml) were cultured in six-well plates and grown for 16–24 h before treatments. After the indicated treatment, the cultures were harvested with ice-cold tris-buffered saline (TBS). Then, cell pellets were re-suspended in protein lysis buffer (0.1M Na_2_HPO_4_, 0.5 M EDTA, 5 M NaCl, 1% Triton X-100, 0.5% sodium deoxycholate, 0.1% SDS, 1 mM PMSF), including protease inhibitor cocktail (Sigma-Aldrich, #11836170001), and phosphatase inhibitor cocktails 2 and 3 (Sigma-Aldrich, P5726 and P0044) that were added according to the manufacturer’s protocol. The re-suspended pellets were kept on ice for 20 min, vortexing every 5 min. The lysates were centrifuged (14000 r.p.m., 10 min), supernatants containing the cellular proteins were collected and stored at −80 °C. Protein concentration was detected by a BCA Protein Assay Kit (ThermoFisher Scientific, #23227; Waltham, MA, USA) using bovine serum albumin as a standard. Protein samples were boiled in SDS-PAGE sample buffer containing 40 mM DDT and resolved by SDS-PAGE (7.5–12% acrylamide). Proteins were then transferred onto a nitrocellulose membrane, which was incubated with the indicated primary antibodies. Secondary antibody conjugates were visualized by SuperSignal West Pico Chemiluminescent Substrate (ThermoFisher Scientific, #34080). Actin was used as loading control. When Ogn secretion to the medium was tested, culture medium was collected, 10-fold concentrated by ultrafiltration, boiled in SDS-PAGE sample buffer containing 40 mM DDT and subjected to immunoblotting analysis. For culture media samples, ponceau staining was used as loading control.

### qRT-PCR and specific PCR primers

B16 cells (4 × 10^5^ cells/ml) or JC TetON LIP cells (2 × 10^5^ cells/ml) were cultured in 24-well plates and grown for 16–24 h before treatments. After the indicated treatment, total RNA was isolated using PerfectPure RNA Cultured Cell Kit (5 Prime, #2900319; Waltham, MA, USA) and reverse transcribed using High Capacity cDNA Reverse Transcription Kit (Applied Biosystems, #4368814; Foster City, CA, USA). Then cDNA was 20-fold diluted and subjected to qRT-PCR analysis, using Absolute Blue qPCR ROX Mix (ThermoFisher Scientific, #AB-4139/A), gene specific primers (designed using Roche ProbeFinder, version 2.5; Roche Diagnostics, Basel, Switzerland) and an appropriate Universal Probes (Roche Diagnostics). The primer sequences were: *Ogn*: 5′-CCATCATTACCAACCAAGAAAGA-3′, 5′-GGTGGTACAGCATCAATGTCA-3′, (probe #21); *C/EBPβ*: 5′-AACCTGGAGACGCAGCAC-3′, 5′-AGCTGCTCCACCTTCTTCTG-3′, (probe #67); *CHOP*: 5′-CTTGAGCCTAACACGTCGATT-3′, 5′-TGCACTTCCTTCTGGAACACT-3′, (probe #21); *p53*: 5′-ATGCCCATGCTACAGAGGAG-3′, 5′-AGACTGGCCCTTCTTGGTCT-3′, (probe #94); *TBP*: 5′-CCAATGACTCCTATGACCCCTA-3′, 5′-CAGCCAAGATTCACGGTAGAT-3′, (probe #51). The amplification was done using Roche LightCycler 480 Real-Time PCR System (Roche Diagnostics). Gene expression level was normalized to TATA box binding protein (*TBP*). The fold change in gene expression compared with mRNA from control cells was calculated using LightCycler 480 Software, version 1.5.0.39. Data are presented as mean±S.D. from three replicates.

### RNAi

B16-F10.9-4 cells (7 × 10^4^ cells/ml) or JC TetON LIP cells (3 × 10^4^ cells/ml) were cultured for 24 h in media lacking penicillin and streptomycin, transfected for 24 h with siRNA pools (Dharmacon, Lafayette, CO, USA) directed against murine c-Jun (L-043776-00-0005), c-Fos (L-041157-00-0005), ATF2 (L-042961-01-0005), ASK1 (M-041179-01-0005), Ogn (L-058799-01-0005), C/EBP*β* (L-043110-00-0005), CHOP (L-062068-00-0005) or p53 (L-040642-00-0005) mRNAs, using DharmaFECT 1 reagent (Dharmacon) according to the manufacturer’s protocol. Then, expression of LIP was induced by Doxy for 16-24 h. ER stress was induced by treating cells for 18–24 h with Tm, BFA or Tg. Cells treated with ON-TARGETplus non-targeting siRNA pool (D-001810-10-20) or siGenome non-targeting siRNA pool (D-001206-14-05), were used as negative control.

### Construction of *Ogn* expression vector and generation of clones stably overexpressing *Ogn*

A cDNA encoding mouse *Ogn* (m*Ogn*) fused to an HA tag at the C-terminus was inserted into pcDNA4/TO (Life Technologies, Waltham, MA, USA) to generate pcHA-m*Ogn*. Murine parental B16-F10 cells (2.5 × 10^5^ cells/ml) were cultured for 24 h in 9 cm plates and then transfected with pcDNA4/TO or pcHA-m*Ogn* using jetPEI (Polyplus-transfection, #101-10 N; IllKirch, France) as a transfection reagent, according to the manufacturer’s protocol. Two days after transfection, the cells were cultured in media containing zeocin (350 *μ*g/ml). Individual colonies were picked up from the plates, grown and tested for Ogn expression by immunoblotting.

### Expression array analysis

B16-F10.9-4 cells (4 × 10^5^ cells/ml) were pretreated with vehicle or Doxy (2 *μ*g/ml), cultured in 24-well plates for 24 h and then treated with Tm (0.25 *μ*g/ml). After 0, 5 and 12 h, the cells were harvested, RNA was purified and subjected to expression array analysis using Affymetrix Mouse Gene 1.0 ST arrays (Santa Clara, CA, USA). The array was performed in triplicate. Analysis of gene expression data, including RMA normalization was performed using the Partek Genomics Suite software, version 6.6 (Partek Inc., St. Louis, MO, USA). A one-way ANOVA (*P*<0.05) was used to select differentially expressed genes. Functional analysis of identified genes was analyzed by DAVID Bioinformatics Resources 6.7 (https://david.ncifcrf.gov) and Ingenuity software (Qiagen Inc., Valencia, CA, USA).

### Statistical analysis

The photographs are representative of at least three replicate experiments unless otherwise stated. All data in the graphs are presented as mean±S.D. from at least three replicates. Statistical analysis was performed using an unpaired, two-tailed Student’s *t*-test. A *P*-value<0.05 was considered as statistically significant. For all figures: *P<0.05, **P<0.01, ***P<0.001.
