# Supplementary material for: C/EBPβ LIP augments cell death by inducing osteoglycin
Source: Cell Death Dis. 2017 Apr 6;8(4):e2733–. doi: 10.1038/cddis.2017.155 (PMC5603828; doi:10.1038/cddis.2017.155)
Supplement: Supplementary Information [file cddis2017155x1.pdf]

Supplementary information

C/EBPβ LIP augments cell death by inducing osteoglycin

Rina Wassermann-Dozorets & Menachem Rubinstein

Supplementary Table 1. List of the most affected genes by a combined treatment of LIP and ER stress, sorted by cellular processes. Log_2 fold induction of transcripts obtained by expression array analysis of RNA from F10.9-4 cells pretreated doxycycline (24 h), followed by tunicamycin for 12 h compared with cells treated with tunicamycin only. N=3.

Supplementary Table 2. List of the most affected genes by a combined treatment of LIP and ER stress. Log_2 fold induction of transcripts obtained by expression array analysis of RNA from F10.9-4 cells treated as described in the legends of supplementary table 1. N=3. The *P*-value for each gene is given in the table. Data for times 0, 5, and 12 h of tunicamycin treatment were submitted to GEO (GEO accession number GSE84155).

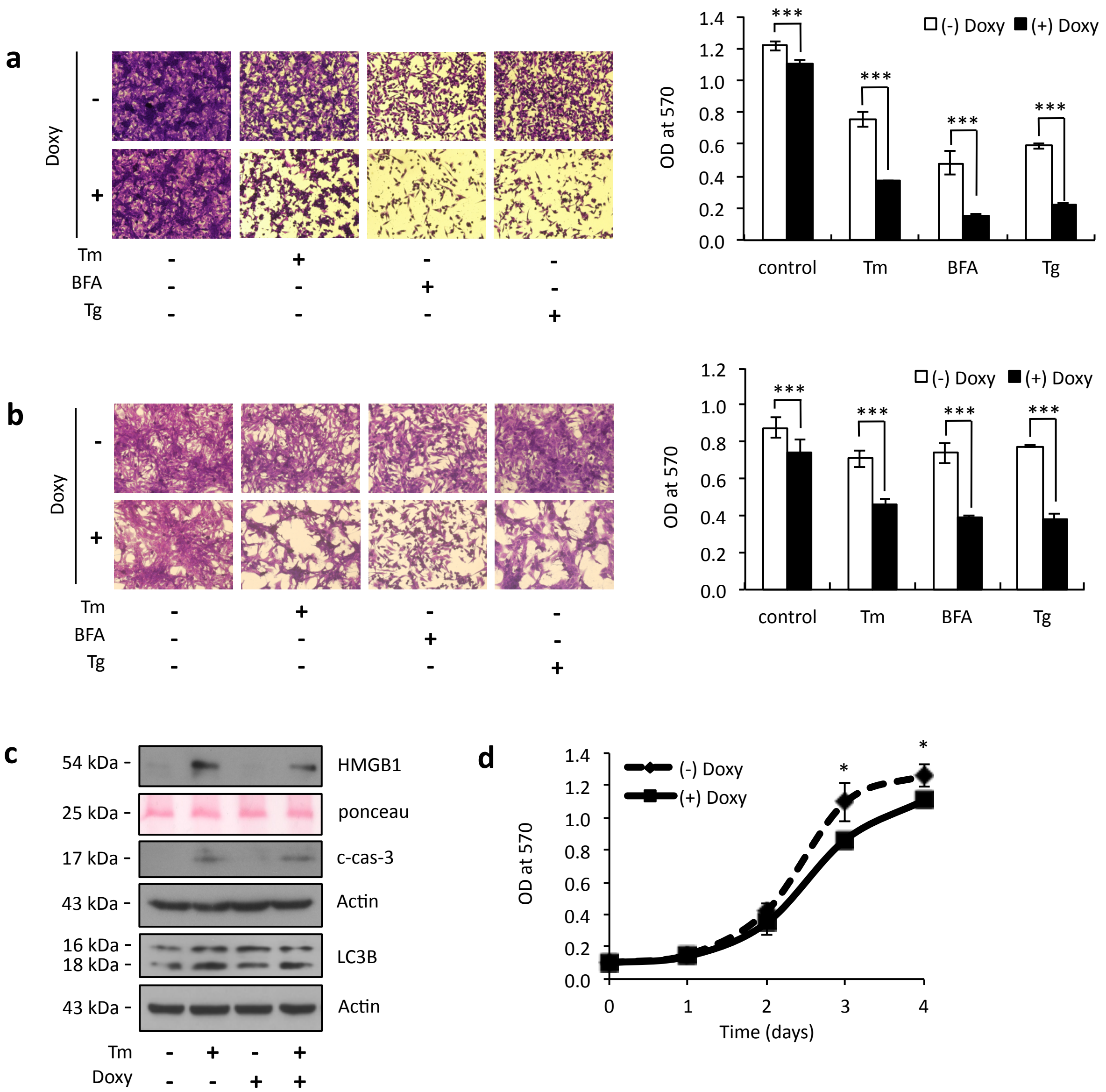

Supplementary Figure 1 LIP augments ER stress-triggered cell death. (**a**) Crystal violet staining of F10.9-4 cells pretreated with vehicle or doxycycline (Doxy), followed by vehicle or tunicamycin (Tm), brefeldin A (BFA) or thapsigargin (Tg). N=3. ****P*<0.001. (**b**) Crystal violet staining of JC TetON LIP cells pretreated with vehicle or Doxy, followed by vehicle or Tm, BFA or Tg. N=3. ****P*<0.001. (**c**) Immunoblot of the indicated proteins in total cell extracts or culture media of F10.9-4 cells pretreated with vehicle or Doxy, followed by vehicle or Tm for 24 h. (**d**) Cell growth rate of F10.9-4 cells pretreated with vehicle or Doxy. N=3. **P*<0.05.

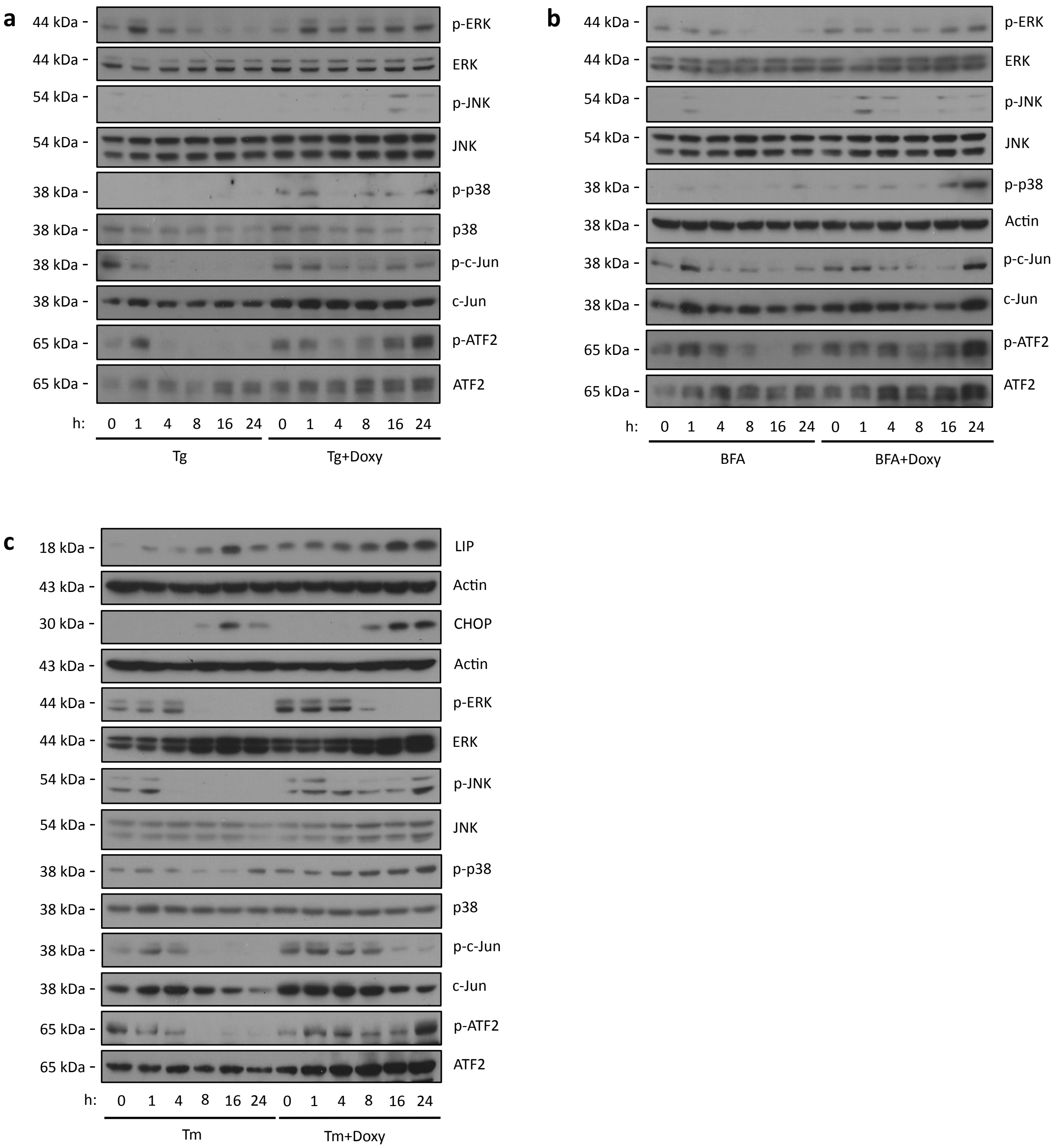

Supplementary Figure 2 LIP induces activation of the MAPK/AP-1 pathway under ER stress. (**a** and **b**) Immunoblot of the indicated proteins in total cell extract of F10.9-4 cells pretreated with vehicle or doxycycline (Doxy), followed by vehicle or thapsigargin (Tg) **a** or brefeldin A (BFA) **b** for the indicated times. (**c**) Immunoblot of the indicated proteins in total cell extract of JC TetON LIP cells pretreated with vehicle or Doxy, followed by vehicle or tunicamycin (Tm) for the indicated times.

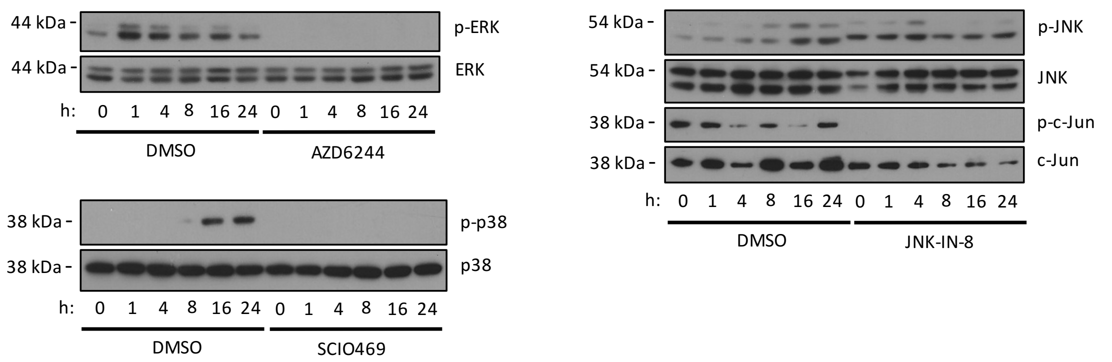

Supplementary Figure 3 Inhibition of MAPK activity with specific inhibitors. Immunoblot of the indicated proteins in total cell extract of F10.9-4 cells pretreated with vehicle or doxycycline (Doxy), followed by vehicle or tunicamycin (Tm) in the presence or absence of the ERK1/2 inhibitor AZD6244, the JNK inhibitor JNK-IN-8 or the p38 inhibitor SCIO469.

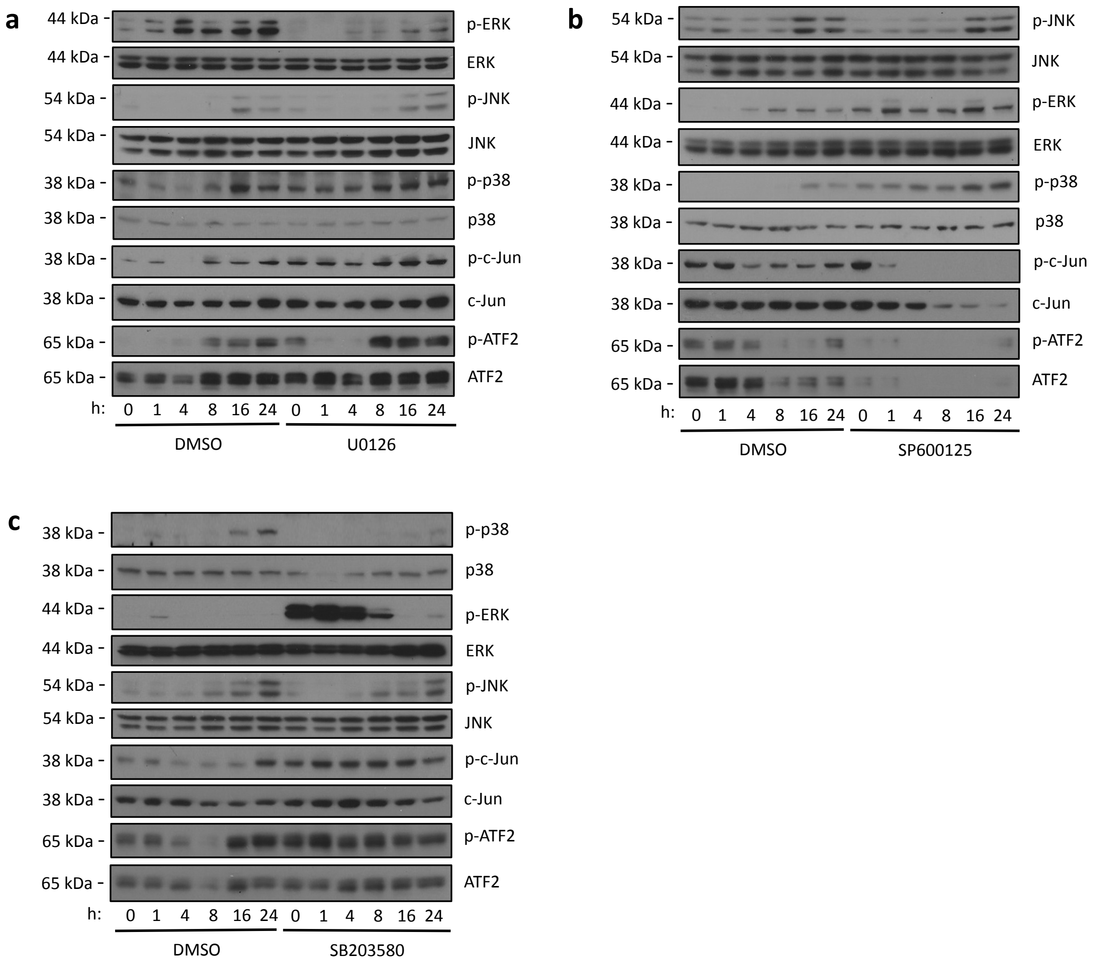

Supplementary Figure 4 Effect of the MAPK inhibitors on activation of the MAPK/AP-1 axis. Immunoblot of the indicated proteins in total cell extract of F10.9-4 cells pretreated with vehicle or doxycycline (Doxy), followed by vehicle or tunicamycin (Tm) in the presence or absence of the ERK1/2 inhibitor U0126 **a**, the JNK inhibitor SP600125 **b**, or the p38 inhibitor SB203580 **c**.

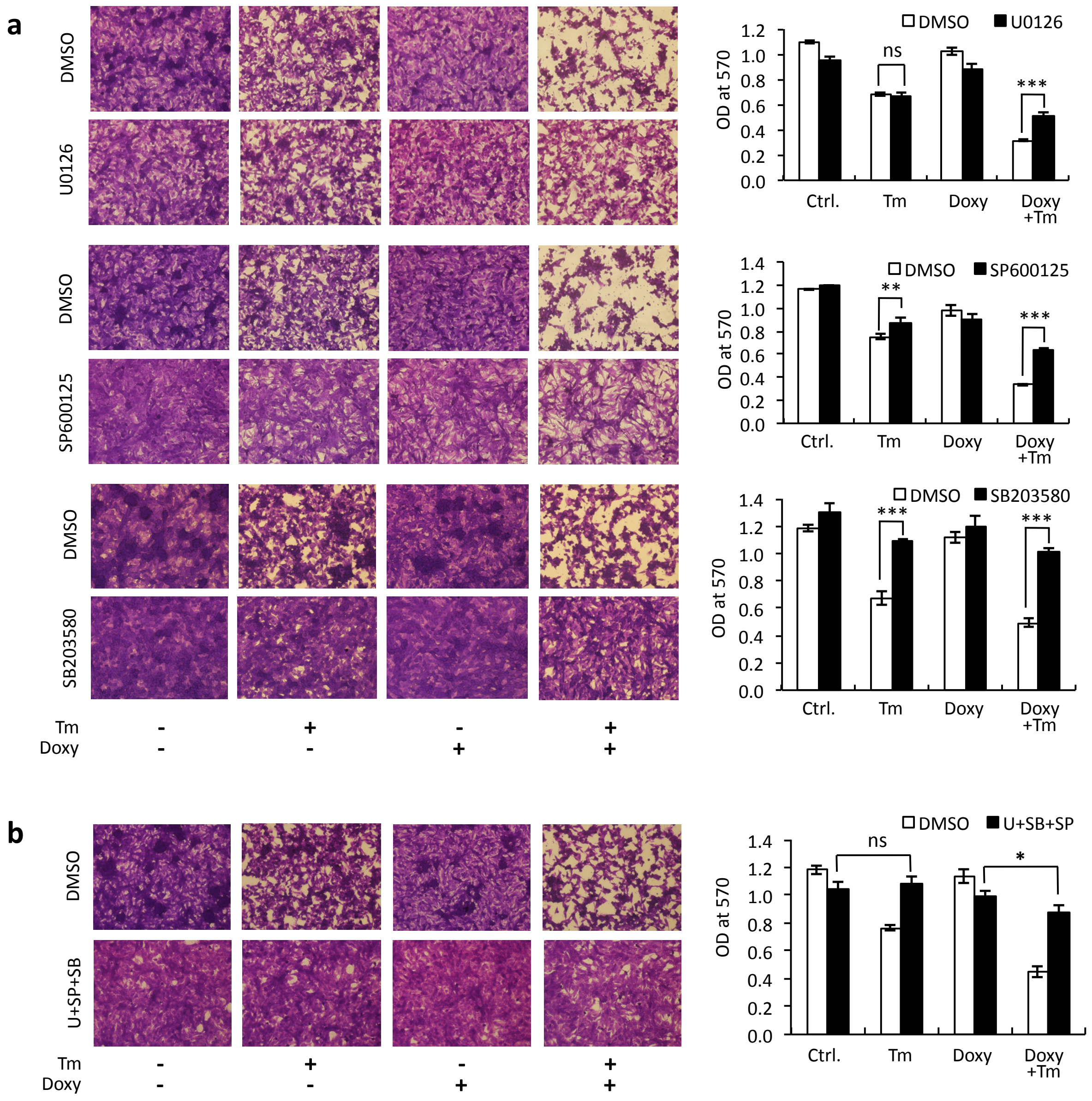

Supplementary Figure 5 LIP augments ER stress-triggered cell death by activating the MAPK pathway. (**a**) Crystal violet staining of F10.9-4 cells pretreated with vehicle or doxycycline (Doxy), followed by vehicle or tunicamycin (Tm), in the presence or absence of the ERK1/2 inhibitor U0126, the JNK inhibitor SP600125 or the p38 inhibitor SB203580. N=3, ***P*<0.01, ****P*<0.001, ns, not significant. (**b**) Crystal violet staining of F10.9-4 cells pretreated with Doxy and Tm as in **a**, in the presence or absence of a combination of the three MAPK inhibitors from **a**. N=4, **P*<0.05, ns, not significant.

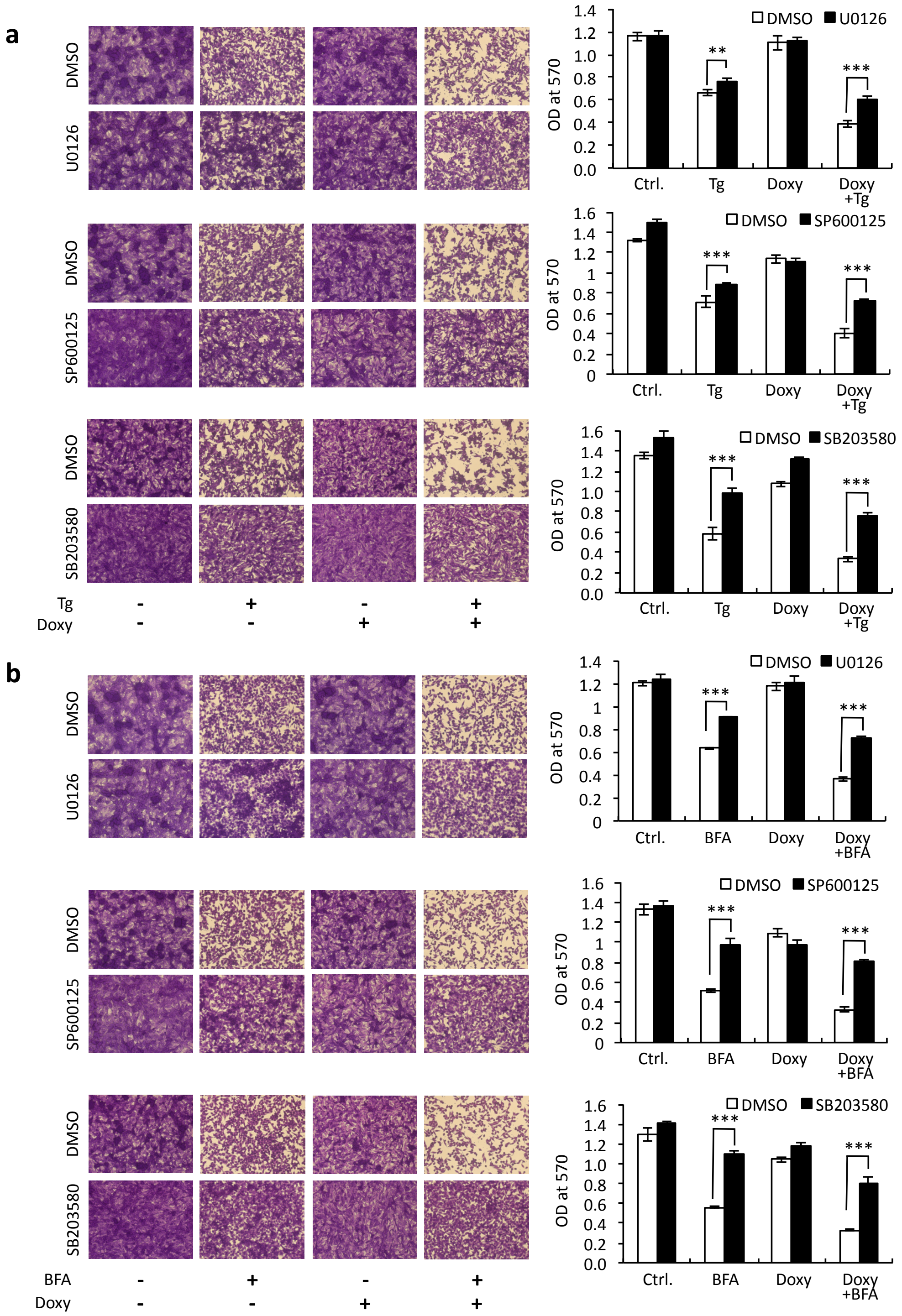

Supplementary Figure 6 LIP augments ER stress-triggered cell death by activating the MAPK pathway. (**a**) Crystal violet staining of F10.9-4 cells pretreated with vehicle or doxycycline (Doxy), followed by vehicle or thapsigargin (Tg), in the presence or absence of the ERK1/2 inhibitor U0126 (20 μM), the JNK inhibitor SP600125 (20 μM), or the p38 inhibitor SB203580 (40 μM). N=2, ***P*<0.01, ****P*<0.001. (**b**) Crystal violet staining of F10.9-4 cells pretreated with vehicle or Doxy, followed by vehicle or brefeldin A (BFA), in the presence or absence of the ERK1/2 inhibitor U0126 (20 μM), the JNK inhibitor SP600125 (20 μM), or the p38 inhibitor SB203580 (20 μM). N=2, ****P*<0.001.

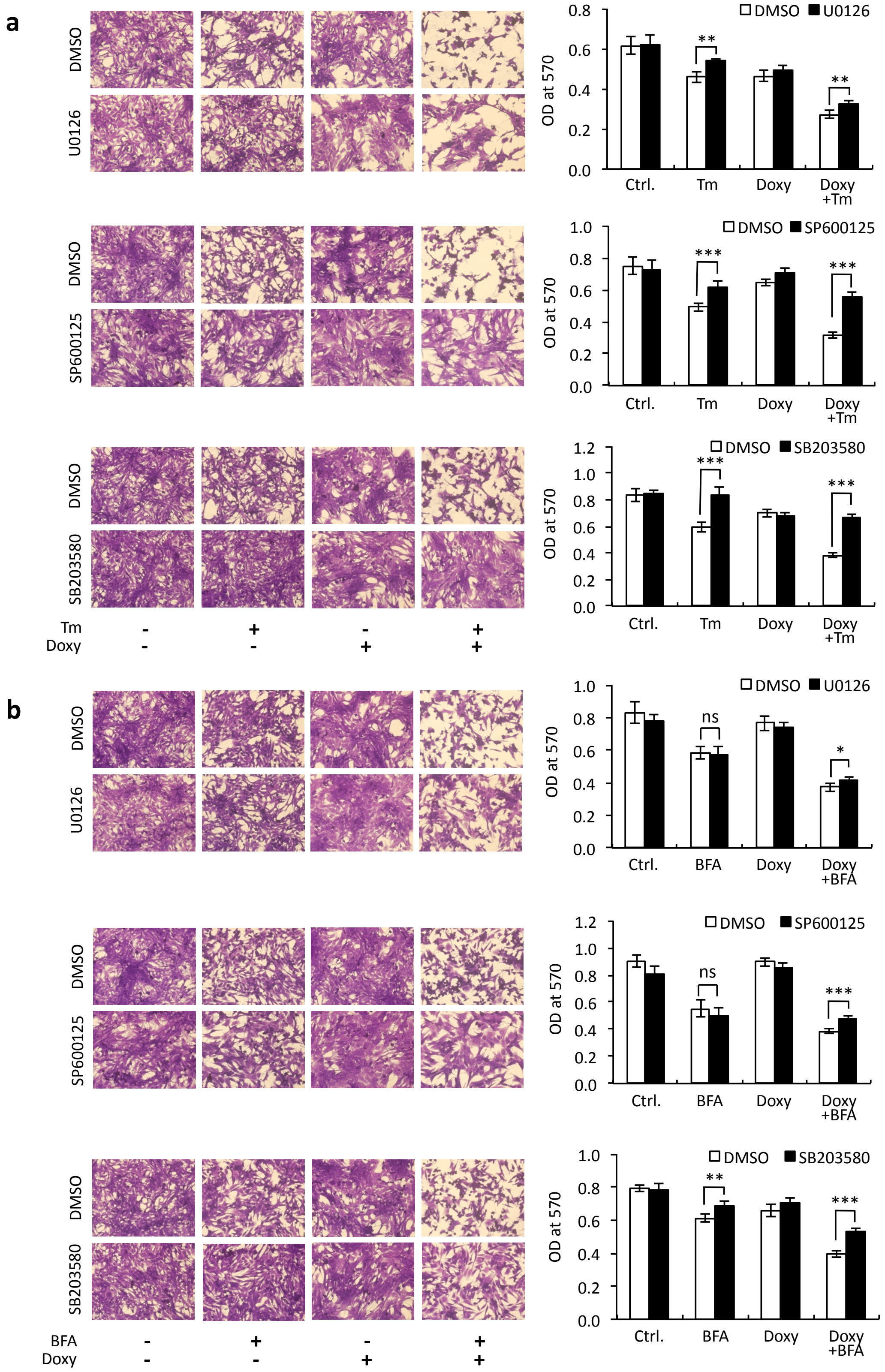

Supplementary Figure 7 LIP augments ER stress-triggered cell death by activating the MAPK pathway. Crystal violet staining of JC TetON LIP cells pretreated with vehicle or doxycycline (Doxy), followed by vehicle or tunicamycin (Tm) **a**, or brefeldin A (BFA) **b**, in the presence or absence of the ERK1/2 inhibitor U0126, the JNK inhibitor SP600125 or the p38 inhibitor SB203580. **P*<0.05, ***P*<0.01, ****P*<0.001, ns, not significant.

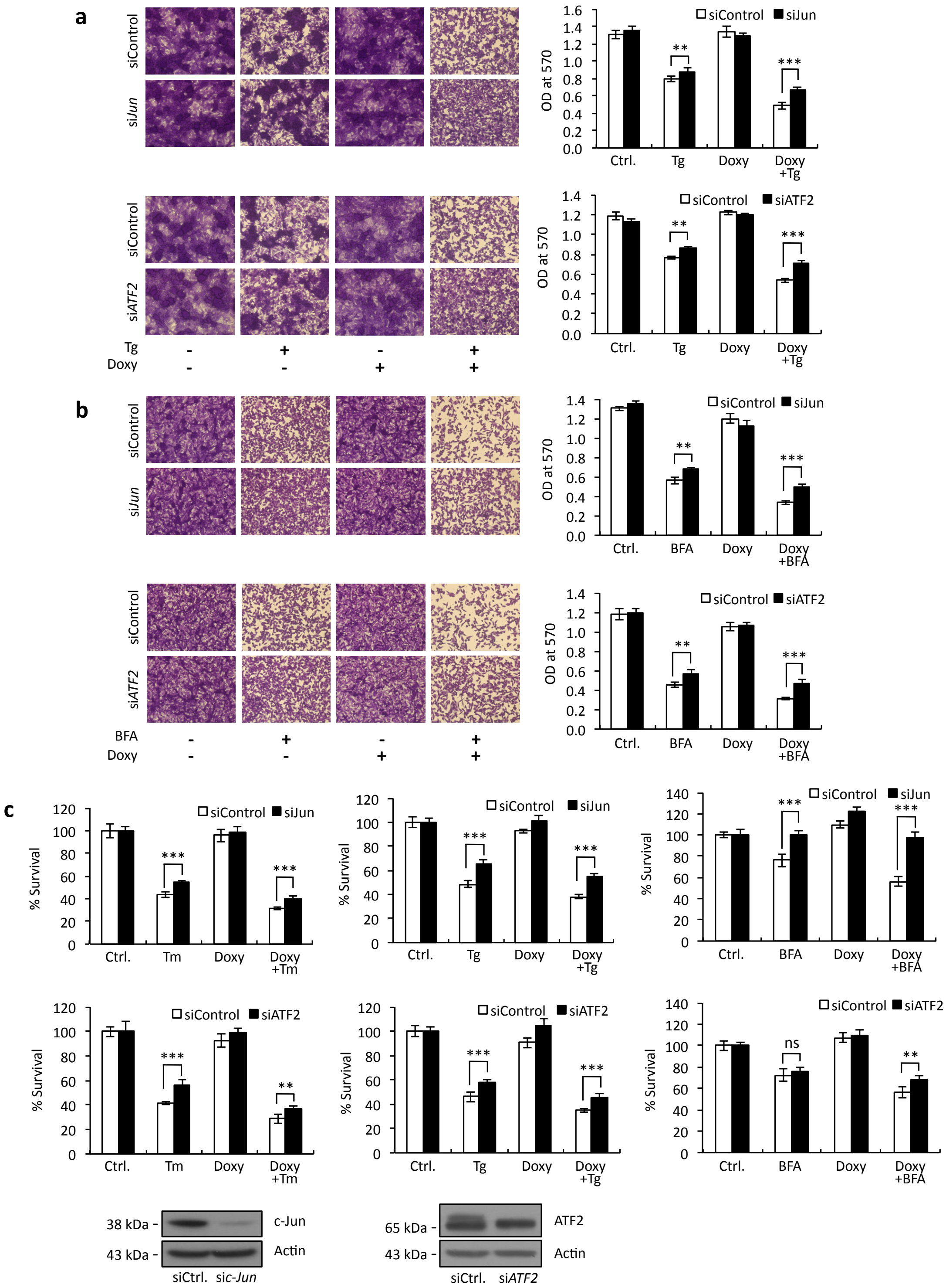

Supplementary Figure 8 The role of AP-1 in augmentation of cell death by LIP. (**a** and **b**) Crystal violet staining of F10.9-4 cells transfected with the indicated siRNA at time=0, treated with vehicle or doxycycline (Doxy) at time=24 h, followed by vehicle or thapsigargin (Tg) **a**, or brefeldin A (BFA) **b** at time=48 h. **P<0.01, ***P<0.001. (**c**) Survival of JC TetON LIP cells transfected with the indicated siRNA at time=0, treated with vehicle or Doxy at time=24 h, followed by vehicle or tunicamycin (Tm) or Tg at time=48 h. N=1-2, **P<0.01, ***P<0.001. Silencing efficacy was evaluated by immunoblotting of total cellular proteins isolated at time=48 h.

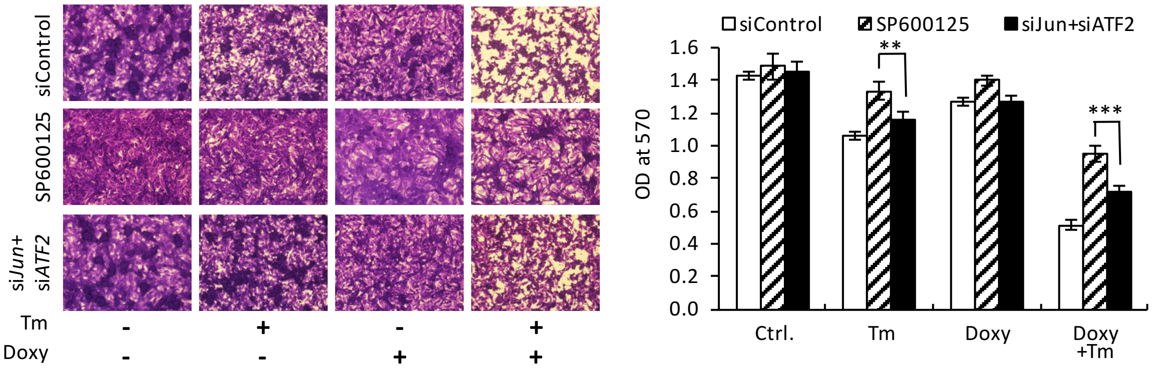

Supplementary Figure 9 The pro-death effect of JNK is not mediated by c-Jun/ATF2 only. Crystal violet staining of F10.9-4 cells transfected with the indicated siRNA at time=0, treated with vehicle or doxycycline (Doxy) at time=24 h, followed by vehicle or tunicamycin (Tm) at time=48 h, in the presence or absence of the JNK inhibitor SP600125. ***P*<0.01, ****P*<0.001.

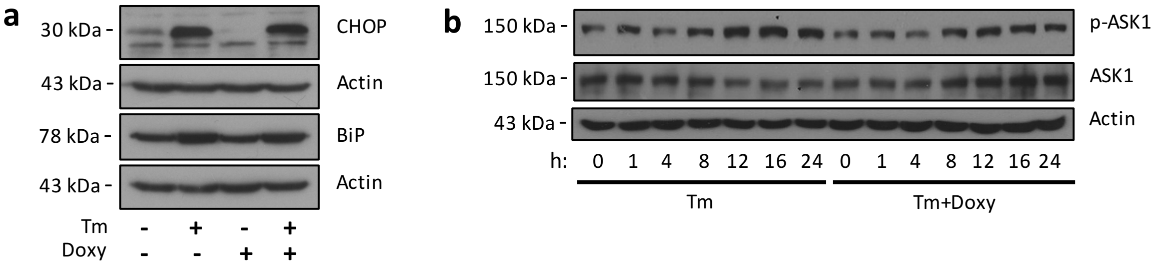

Supplementary Figure 10 The UPR is not involved in LIP augmentation cell death. (**a**) Immunoblot of the indicated proteins in total cell extract of F10.9-4 cells pretreated with vehicle or doxycycline (Doxy), followed by vehicle or tunicamycin (Tm) for 24 h. (**b**) Immunoblot of the indicated proteins in total cell extract of F10.9-4 cells pretreated with vehicle or Doxy, followed by vehicle or Tm for the indicated times.

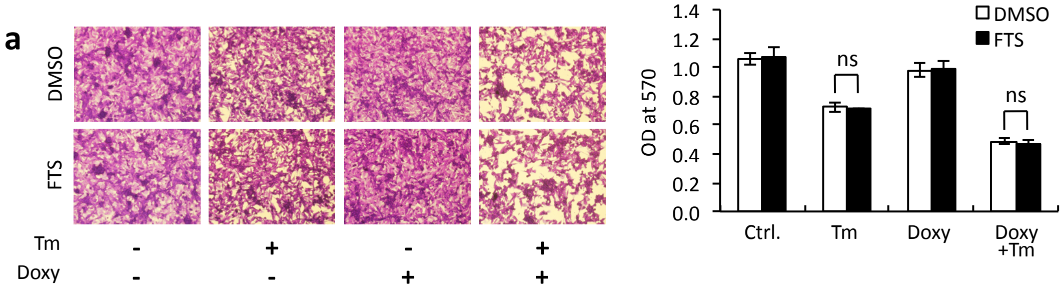

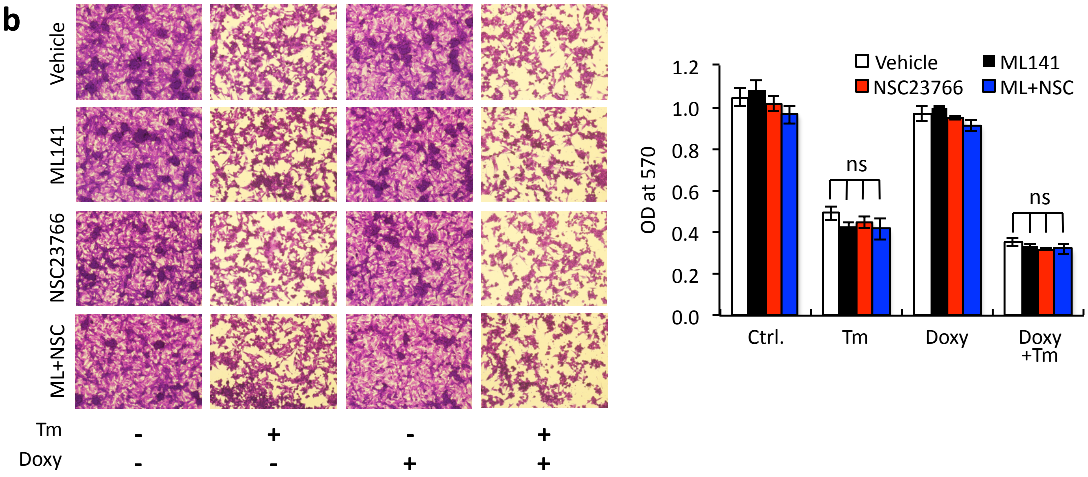

Supplementary Figure 11 Small GTPases from the Ras/Rho family are not involved in LIP augmentation of cell death. (**a**) Crystal violet staining of F10.9-4 cells pretreated with vehicle or doxycycline (Doxy), followed by vehicle or tunicamycin (Tm), in the presence or absence of the Ras inhibitor FTS. N=2, ns, not significant. (**b**) Crystal violet staining of F10.9-4 cells pretreated with vehicle or Doxy, followed by vehicle or Tm, in the presence or absence of the Rac1 inhibitor NSC23766 or the CDC42 inhibitor ML141. N=2, ns, not significant.

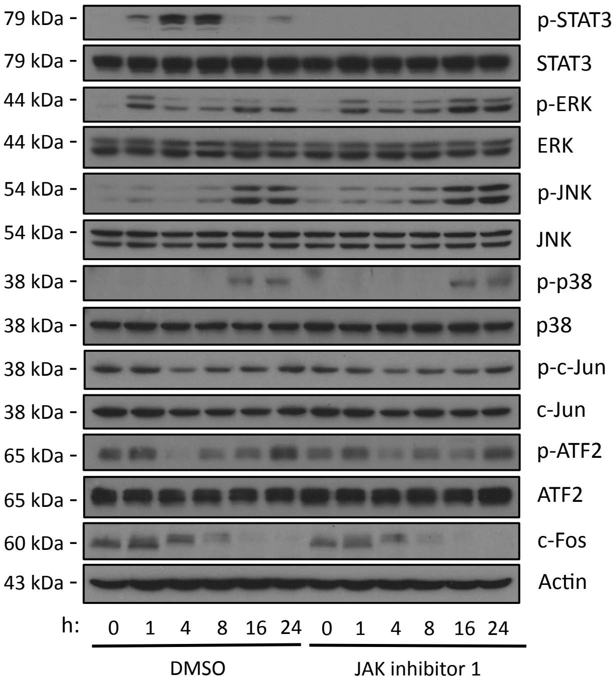

Supplementary Figure 12 LIP activates the MAPK/AP-1 pathway independently of JAK/STAT3. Immunoblot of the indicated proteins in total cell extract of F10.9-4 cells pretreated with vehicle or doxycycline (Doxy), followed by vehicle or tunicamycin (Tm), in the presence or absence of the JAK inhibitor 1.

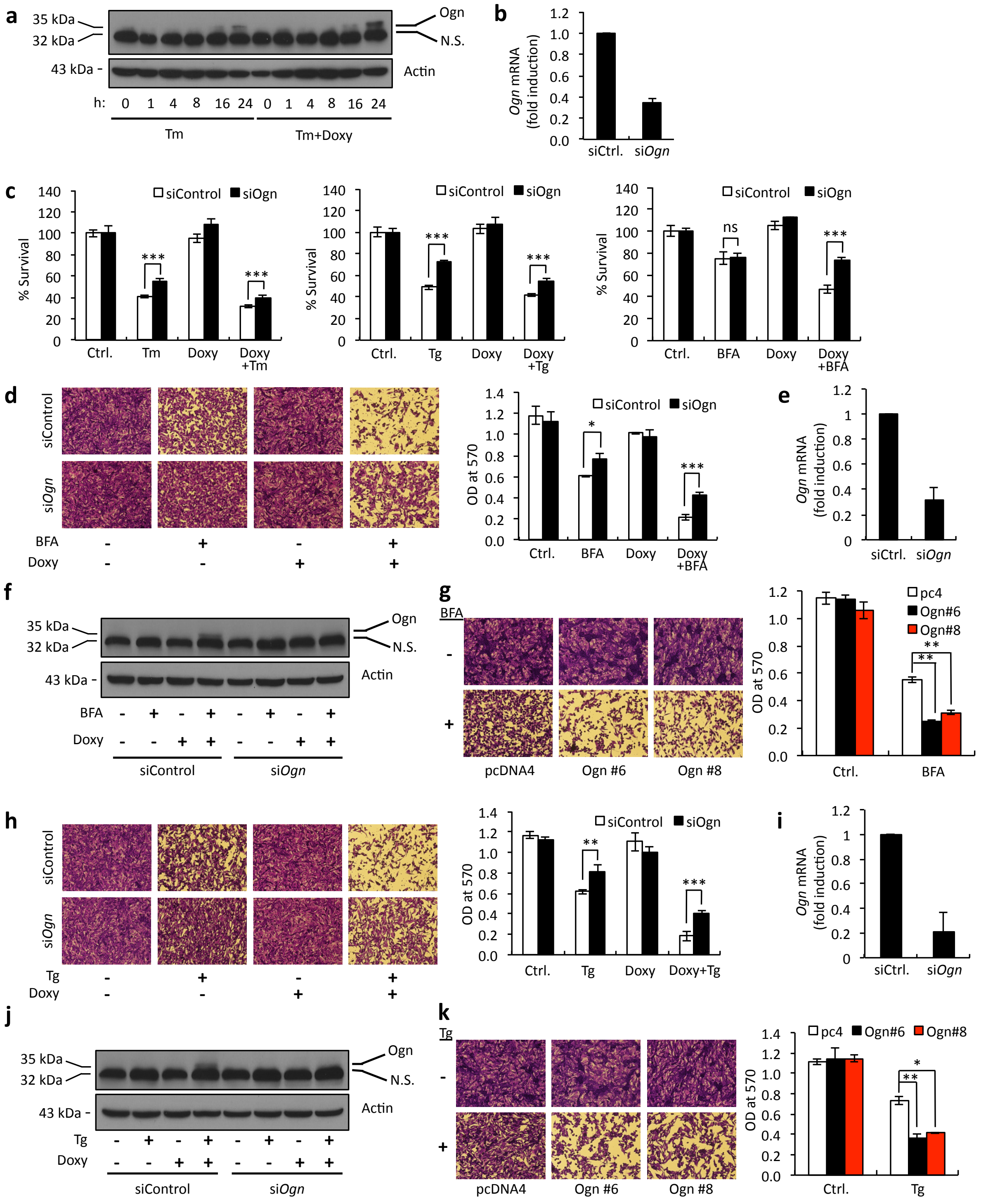

Supplementary Figure 13 Osteoglycin augments ER stress-triggered cell death. (**a**) Immunoblot of Ogn in total cell extract of JC TetON LIP cells pretreated with vehicle or doxycycline (Doxy), followed by vehicle or tunicamycin (Tm) for the indicated times. N=2, N.S., non-specific band. (**b**) qRT-PCR of *Ogn* mRNA in extracts JC TetON LIP cells transfected at time=0 with control siRNA or *Ogn*-specific siRNA. Total mRNA was isolated at time=48 h. (**c**) Survival of JC TetON LIP cells transfected at time=0 with control siRNA or *Ogn*-specific siRNA, treated with vehicle or Doxy at time=24 h, followed by vehicle or Tm or thapsigargin (Tg) at time=48 h. ****P*<0.001. (**d** and **h**) Crystal violet staining of F10.9-4 cells transfected

with control siRNA or *Ogn*-specific siRNA at time=0, treated with vehicle or Doxy at time=24 h, followed by vehicle or brefeldin A (BFA) **d** or Tg **h** at time=48 h. N=2, *P<0.05, **P<0.01, ***P<0.001. (**e** and **i**) qRT-PCR of *Ogn* mRNA in extracts F10.9-4 cells transfected at time=0 with control siRNA or *Ogn*-specific siRNA. Total mRNA was isolated at time=48 h before BFA **e** or Tg **i** treatment. (**f** and **j**) Immunoblot of Ogn in total cell extracts of F10.9-4 cells treated as described in **d** and **h**, respectively. N.S., non-specific band. (**g** and **k**) Crystal violet staining of B16-F10 clones expressing Ogn and control pcDNA4-TO-transfected clone, treated with vehicle or BFA **g** or Tg **k** and then stained. N=2, *P<0.05, **P<0.01.

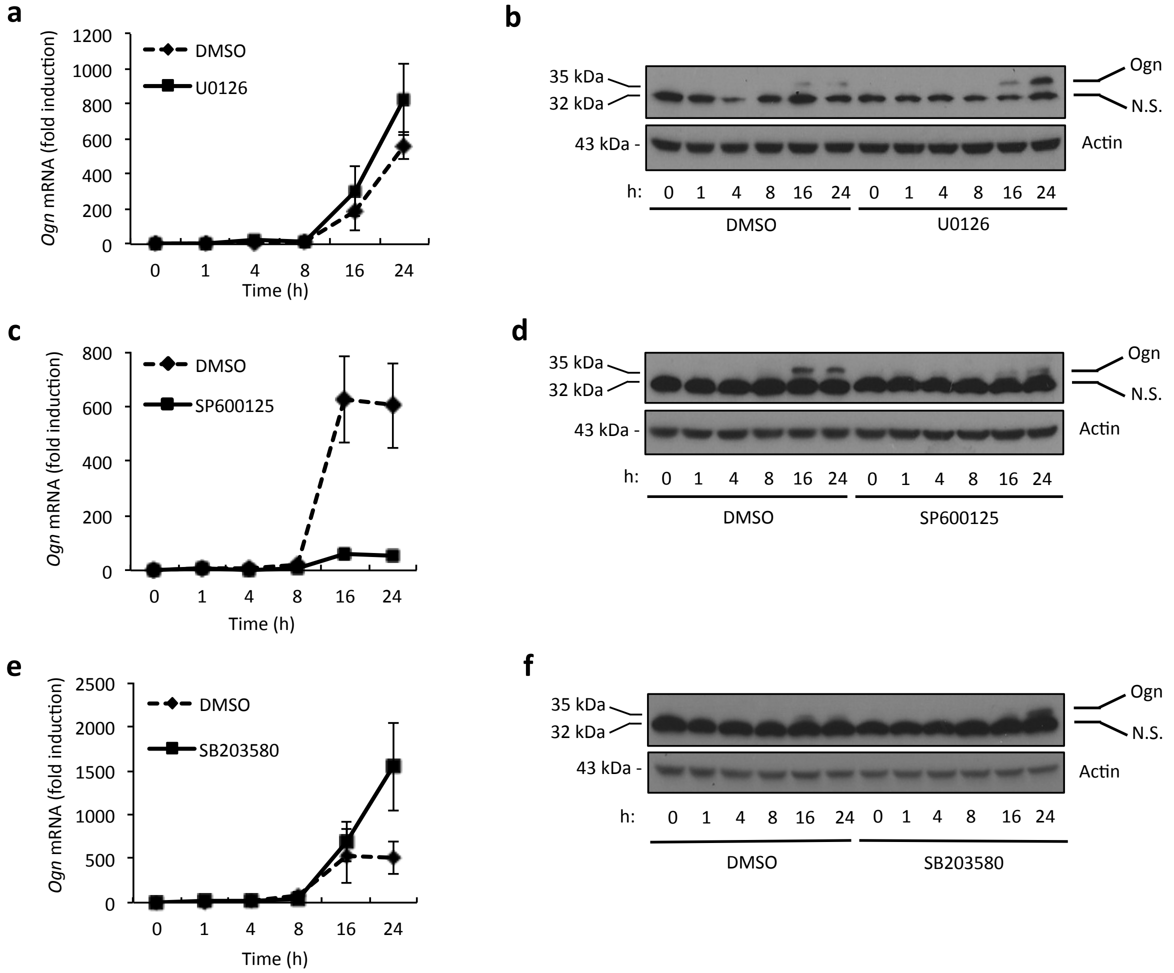

Supplementary Figure 14 Ogn expression is regulated by the MAPK/AP-1 axis. (**a, c, e**) qRT-PCR of *Ogn* mRNA isolated from F10.9-4 cells pretreated with vehicle or doxycycline (Doxy), followed by vehicle or tunicamycin (Tm) for the indicated times, in the presence or absence of the ERK1/2 inhibitor U0126 **a**, the JNK inhibitor SP600125 **c** or the p38 inhibitor SB203580 **e**. (**b, d, f**) Immunoblot of Ogn in total cell extracts of F10.9-4 cells treated as described in **a, c, e**. N.S., non-specific band.

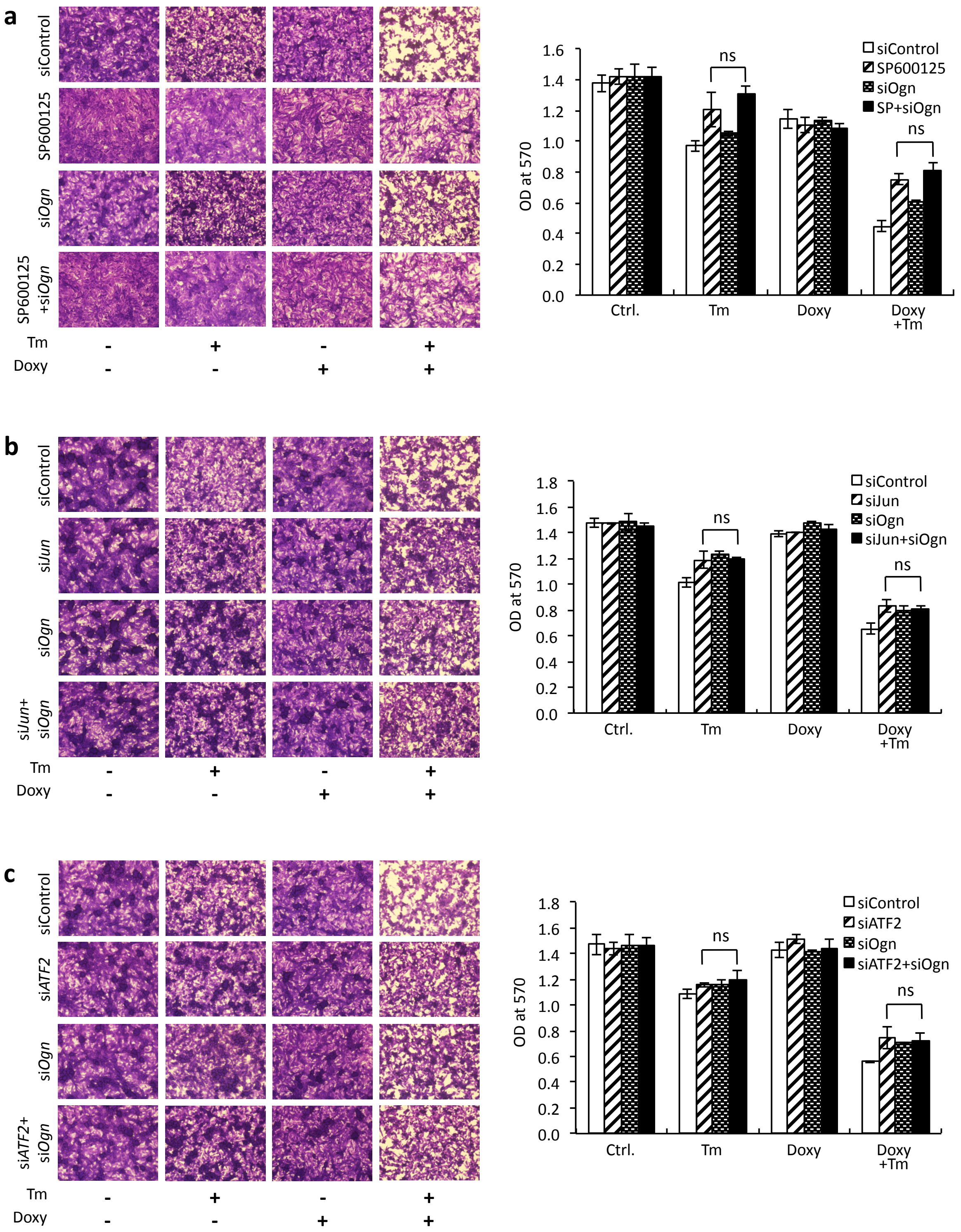

Supplementary Figure 15 Ogn is mainly regulated through the JNK/c-Jun axis. (**a**) Crystal violet staining of F10.9-4 cells transfected with the indicated siRNA at time=0, treated with vehicle or doxycycline (Doxy) at time=24 h, followed by vehicle or tunicamycin (Tm) at time=48 h, in the presence or absence of the JNK inhibitor SP600125. N=2, ns, not significant. (**b** and **c**) Crystal violet staining of F10.9-4 cells transfected with the indicated siRNA at time=0, treated with vehicle or Doxy at time=24 h, followed by vehicle or Tm at time=48 h. N=2, ns, not significant.

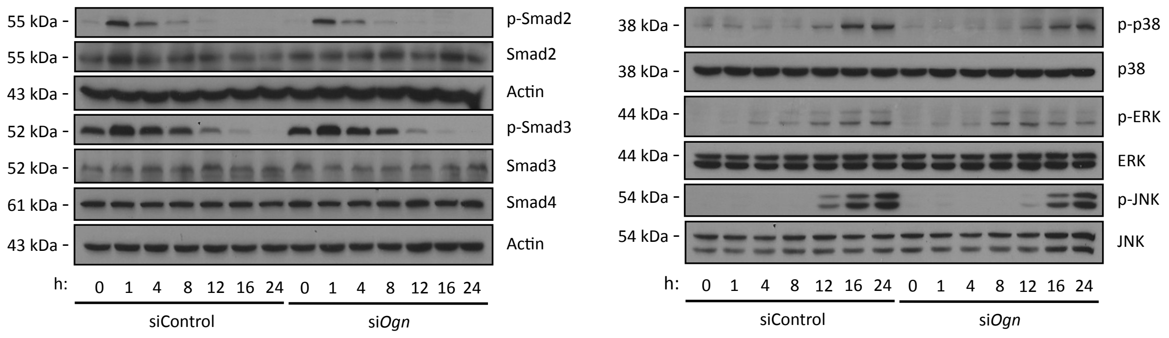

Supplementary Figure 16 Ogn does not signal through the TGFβ pathway. Immunoblot of the indicated proteins in total cell extract of F10.9-4 cells transfected with control siRNA or *Ogn*-specific siRNA at time=0, treated with vehicle or doxycycline (Doxy) at time=24 h, followed by vehicle or tunicamycin (Tm) for the indicated times.
